# Outbreaks of meningococcal meningitis in non-African countries over the last 50 years: a systematic review

**DOI:** 10.7189/jogh.09.010411

**Published:** 2019-06

**Authors:** Femke van Kessel, Caroline van den Ende, Anouk M Oordt-Speets, Moe H Kyaw

**Affiliations:** 1 Pallas Health Research and Consultancy, Rotterdam, the Netherlands; 2 Sanofi Pasteur, Swiftwater, Pennsylvania, USA

## Abstract

**Background:**

Meningococcal disease is caused by the bacteria *Neisseria meningitidis*, leading to substantial mortality and severe morbidity; with serogroups A, B, C, W135, X and Y most significant in causing disease. An outbreak is defined as multiple cases of the same serogroup occurring in a population over a short time-period. A systematic review was performed to gain insight into outbreaks of meningococcal disease and to describe the temporal pattern over the last 50 years in non-African countries.

**Methods:**

PubMed and EMBASE were searched for English-language publications on outbreaks of meningococcal disease in non-African countries between January 1966 and July 2017, with an additional grey literature search. Articles and reports were considered eligible if they reported confirmed meningococcal outbreak cases, included the region, number of cases, and the start and end dates of the outbreak. Data on outbreaks was stratified by geographical region in accordance with the World Health Organization (WHO) regional classification, and case-fatality rates (CFRs) were calculated.

**Results:**

Of the identified publications, 3067 were screened and 73 included, reporting data from 83 outbreaks. The majority of outbreaks were identified in the regions of the Americas (41/83 outbreaks), followed by the European region (30/83 outbreaks). In each of the Western Pacific, Eastern Mediterranean, and South-East Asian regions there were <10 outbreaks reported. The predominant serogroup in the majority of outbreaks was serogroup C (61%), followed by serogroup B (29%), serogroup A (5%) and serogroup W135 (4%). Outbreaks showed a peak in the colder months of both the Northern and Southern Hemispheres. Of the 54 outbreaks where CFR was calculable for all outbreak cases, it ranged from 0%-80%.

**Conclusions:**

These data present a retrospective view of the patterns for meningococcal disease outbreaks in non-African countries, and provide valuable data for monitoring future changes in disease epidemiology and informing preventive measures.

Meningococcal disease is caused by invasive infection by *Neisseria meningitidis* bacteria. It is an unpredictable, contagious disease that leads to substantial mortality and severe morbidity, with long-term sequels including limb loss, hearing loss, and brain damage [1]. There are two clinical forms of meningococcal disease; meningococcal meningitis, inflammation of the meninges, and meningococcal septicemia, systemic infection of the blood with subsequent shock and disseminated intravascular coagulation, which has a much higher fatality rate [2]. The number of cases of meningococcal meningitis worldwide has been estimated to be at least 1.2 million per annum, with a round 135 000 deaths [1]. With the use of prompt antibiotic treatment in recent years, the case-fatality rate (CFR) has reduced to 10%-20% from 70%-85% in pre-antibiotic eras [1,3], with serious and long-term sequelae occurring in 10%-20% of survivors [4]. Although meningococcal disease frequently occurs as scattered, apparently unrelated cases or in small outbreaks, in some regions this endemic situation may alternate with devastating, unpredictable epidemics. This is the case in the African Meningitis Belt (stretching from Ethiopia to Senegal), which has the highest incidence of meningococcal disease in the world [1,3,5]. The incidence of meningococcal disease is generally highest in infants under one year old, with another peak in adolescents and young adults [3,6].

Thirteen serogroups of *N. meningitidis* have been characterized, with serogroups A, B, C, W135, X and Y most significant in terms of causing disease [1]. The serogroup responsible for meningococcal disease cases varies over time and by geographical area [7]. The prevention of meningococcal disease is through the use of vaccination, either as routine immunization, or vaccination at times of outbreaks or epidemics. Polysaccharide vaccines against serogroups A, C, Y and W135 have been available since the 1970s and 1980s, however these elicit a poor, short-lived, immunological response, particularly in those less than two years of age [7]. In more recent years, conjugated vaccines have been introduced, these are available as monovalent (A or C) or tetravalent (A, C, Y, W135) vaccines [7]. Only since 2013 have vaccines against serogroup B become available, due to challenges in identifying target antigens [8]; since 2015 they have been used in routine immunization of infants in the UK [9], and for at-risk individuals aged ≥10 years in the US [10]. Immunization protocols against meningococcal disease vary by country [3,11,12], driven by local patterns of disease.

In the African meningitis belt, the WHO definition of a meningococcal epidemic is >100 cases/100 000 population/yeaer [5]. Outside the meningitis belt, where meningococcal disease occurs less frequently, an outbreak is defined as multiple cases of the same serogroup occurring in a defined population over a short time period [11]. Meningococcal disease outbreaks can have a significant burden on health care utilization and costs, in both the short- and long-term [13-17]. Limited data however are currently available on meningococcal outbreaks, which are very useful in helping to understand the burden of meningococcal disease on patients and society, and to inform public health strategies. The aim of this systematic review is to describe the temporal pattern and nature of outbreaks of meningococcal disease reported over the last 50 years. As the disease pattern in the meningitis belt is very different from other countries, with frequent epidemics, the current review focuses on meningococcal outbreaks in non-African countries.

## METHODS

The study protocol for this systematic review was registered to PROSPERO, CRD42017074957 [18], and followed the Preferred Reporting Items for Systematic reviews and Meta-Analyses (PRISMA) guidelines.

### Identification of eligible publications

The PubMed and EMBASE databases were searched for articles on outbreaks of meningococcal disease published between January 1966 and July 2017 (search terms listed in Table S1 in **Online Supplementary Document**); an additional search of the grey literature using the terms ‘meningococcal’ and ‘outbreak’ was conducted in September 2017 which included the websites of ProMED mail, the World Health Organization (WHO), the Centers for Disease Control and Prevention (CDC) and the European Centre for Disease Prevention and Control (ECDC) (Table S1 in **Online Supplementary Document**).

Articles and reports were considered eligible if they were written in English language, reported clinically or laboratory confirmed meningococcal outbreak cases (as defined in each identified study), included the region of outbreak, number of cases, and the start and end date of the outbreak. Exclusion criteria included: non-relevant publication types (such as expert opinions, letters to the editor, editorials, comments, narrative reviews, or case reports), modeling studies that did not provide original data, genetic carriage studies, animal studies, studies on outbreaks in the African region, studies which focused on a subgroup of outbreak cases, studies where it was unclear whether all outbreak cases were described, general incidence/prevalence studies without a focus on outbreaks, or studies describing a meningococcal epidemic. To prevent inclusion of multiple publications on the same outbreak, only the most recent or most complete publication was selected. The reference list of meta-analyses and good quality systematic reviews were also checked for possibly missed relevant articles.

### Study screening

Screening of peer-reviewed publications was conducted first on the title and abstract, followed by screening of the full-text article. The first 30% of titles and abstracts, and the first 10% of the full-text articles were screened in duplicate, the results were compared and discussed and any disagreements were adjudicated by a third, and if necessary fourth, researcher until consensus was reached. During the screening process there was less than 5% discrepancy between the two researchers.

Results from ProMED mail were first screened based on title and abstract, followed by full-text screening, as with the peer-reviewed articles. All search hits from the other websites were fully screened, except for the WHO website, for which only the first page of search results was screened in addition to an overview page mentioning all meningococcal outbreaks worldwide in the last 50 years.

### Data extraction

Data from peer-reviewed studies were extracted into a table in MS Word (Microsoft Inc, Seattle, WA) by one researcher, in close collaboration with a second researcher. A separate data extraction table was used for the grey literature. Information identified from the studies included study design, study characteristics (ie, country, study population and setting, outbreak period, source of outbreak), case detection and definition, and data on the meningococcal outbreak (ie, number of cases, age/gender, *N. meningitidis* serogroup, outcomes of the outbreak, control intervention). All data extraction tables were then reviewed by a second researcher. As most studies were not of a classical design suited to appraisal using existing checklists, no checklists were used to assess the quality of the articles or to calculate a total quality score. Nevertheless, some articles were excluded because of major limitations in their design or reporting of outbreaks, and included in the category ‘Description of outbreak unclear or incomplete’.

### Outcomes

Data on outbreaks were visualized by timelines and stratified by geographical region in accordance with the WHO regional classification (the region of the Americas (stratified for North and South American countries), South-East Asia region, European region, Eastern Mediterranean region (including Israel), and the Western Pacific region). The identified dates of each outbreak allowed assessment of the seasonal pattern of outbreaks by assessing the number of outbreaks that occurred each month split by Northern and Southern Hemisphere, and the occurrence of outbreaks in 10-year periods (1966-1975, 1976-1985, 1986-1995, 1996-2005, 2006-July 2017). Where possible, CFRs were calculated for each included outbreak as the number of deaths/number of cases.

## RESULTS

**Figure 1:**
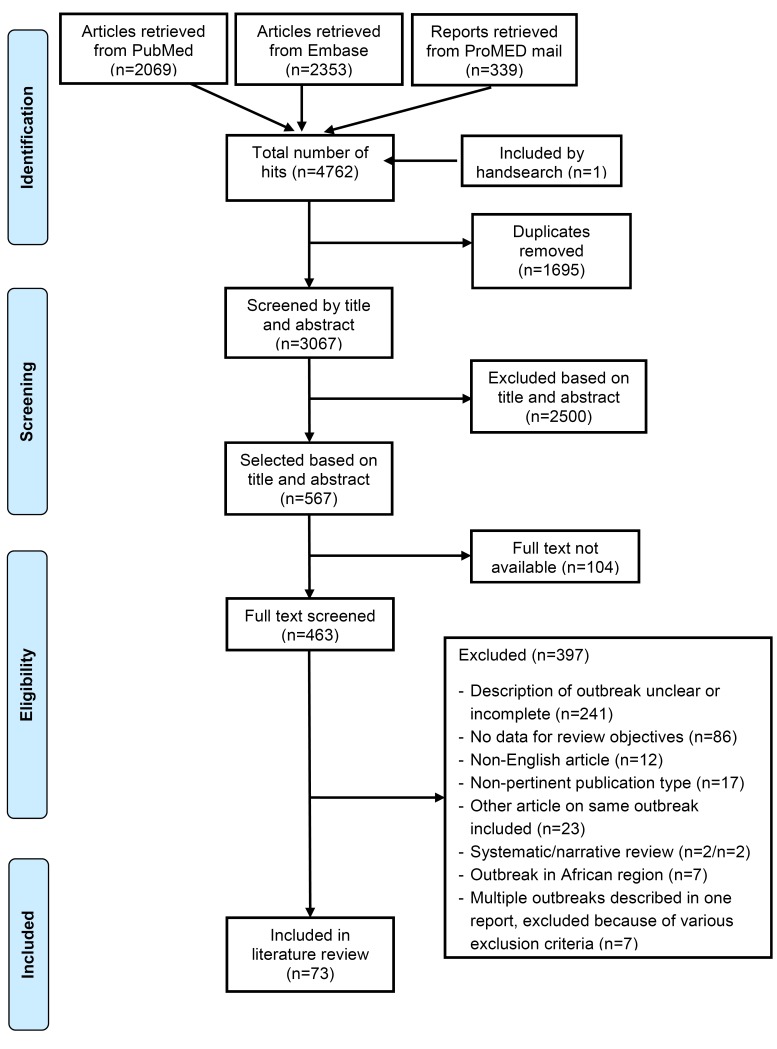
Summary of study selection process.

After removal of duplicates from the 4762 publications identified from PubMed, EMBASE and ProMED mail, 3067 publications were screened and 73 were included in this review; 66 from the peer-reviewed literature, 7 from ProMED mail (**Figure 1**). Details of the included studies are included in Table S2 in **Online Supplementary Document**; the 73 included studies reported outbreak data from 83 meningococcal outbreaks (**Table 1**).

**Table 1:** Summary of outbreaks of meningococcal disease in non-African countries by region

Country (region)	Outbreak period	Number of cases	Predominant serogroup	CFR (deaths/cases)*
**Region of the Americas**				
Canada (Eastern Ontario/Western Quebec) [19]	December 1991 – January 1992	10	C	50% (5/10)
Canada (Kitchener-Waterloo, Ontario) [20]	3 December 1997 – 4 January 1998	7	C	29% (2/7)
Canada (Edmonton, Alberta) [21]	December 1999 – April 2001	61	C†	[4% (2/56)]
Canada (Abbotsford, British Columbia) [22]	December 2000 – March 2001	5	C	40% (2/5)
Canada (Toronto, Ontario) [23]	Early May – mid July 2001	6	C	33% (2/6)
Canada (Abbotsford, British Columbia) [22]	October 2001 – December 2001	4	C	75% (3/4)
Canada (Abbotsford, British Columbia) [22]	September – December 2004	7	C	71% (5/7)
Canada (Nova Scotia) [24]	1-11 February 2015	2	B	50% (1/2)
USA (United States Army Infantry Training Center, Fort Lewis, Washington) [25]	14 December 1970 – June 1971	15	C	[100% (3/3)]
USA (Los Angeles area, San Francisco area) [26]	1-13 April 1974	5	B	NR
USA (Washington DC) [27]	9-16 May 1979	3	B	0% (0/3)
USA (Vermont) [28]	15 February 1984	13	C	0% (0/13)
USA (Rockbridge County, northwestern Virginia) [29]	15-16 February 1986	5	C	0% (0/5)
USA (Benton, Chelan, Douglas, Grant, Klickitat, and Yakima) [30]	12 January – August 1989	28	C	NR‡
USA (Santa Clara County, California) [31]	27 January – 7 February 1989	5	C	0% (0/5)
USA (Benton, Chelan, Douglas, Grant, Klickitat, and Yakima) [30]	September 1989 – August 1990	12	C	NR‡
USA (Benton, Chelan, Douglas, Grant, Klickitat, and Yakima) [30]	September 1990 – August 1991	5	C	NR‡
USA (region NR) [32]	8 February 1991 – 0 April 1992	9	C	33% (3/9)
USA (Iowa City, Iowa) [33]	23 October – 15 December 1992	5	C	0% (0/5)
USA (Los Angeles County Men’s Jail system, California) [34]	1 January – 31 March 1993	11	C§	NR
USA (Grayson County, North Texas) [35]	24 February – 21 March 1993	7	C	14% (1/7)
USA (Connecticut) [36]	May 1993	3	A	0% (0/3)
USA (region NR) [37]	11-18 February 1995	6	B	0% (0/6)
USA (Florida) [38]	8 July – August 1995	5	B	20% (1/5)
USA (Florida) [38]	2-10 December 1997	3	B	33% (1/3)
USA (Putnam County, Florida) [39]	12 December 1998 – 28 December 1999	12	C	17% (2/12)
USA (Chicago) [40]	6-15 October 2003	6	C	50% (3/6)
USA (central Brooklyn, New York) [41]	1 November 2005 – 30 November 2006	23	C	30% (7/23)
USA (northeastern Oklahoma) [42]	10 March-31 March 2010	7	C#	[40% (2/5)]
USA (California) [43]	March-November 2013	5	B	NR
USA (New Jersey) [44]	25 March 2013 – 10 March 2014	9	B	11% (1/9)
USA (Eugene, Oregon) [45,46]	13 January – 19 May 2015	7	B	14% (1/7)
USA (Providence College, Rhode Island) [47]	2-5 February 2015	2	B	0% (0/2)
USA (Santa Clara University, California) [48]	31 January – 2 February 2016	3	B	0% (0/3)
Brazil (Rio Verde city) [49]	June-August 2008	22	C#	[31% (5/16)]
Brazil (Trancoso, Seguro, Bahia State) [50]	21-26 October 2009	9	C	67% (6/9)
Brazil (Cosmópolis and Săo José dos Campos, Săo Paulo State) [51]	29 March – 30 June 2010	18	C	17% (3/18)
Brazil (Cosmópolis and Săo José dos Campos, Săo Paulo State) [51]	10 July – 8 August 2010	13	C	46% (6/13)
Chile (Metropolitan Region, includes Santiago) [52]	January – November 2013	46	W135	NR
Mexico (Tijuana) [53]	30 January – 30 March 2013	19	C	37% (7/19)
Trinidad and Tobago (El Socorroi, San Juan and Claxton Bay) [54]	26 September – 10 October 1998	21	B¶	[57% (8/14)]
**European region**				
Belgium, Denmark, Germany, The Netherlands (region NR) [55]	9 May – 24 December 1997	11	C	9% (1/11)
Czech Republic (Olomouc and Bruntal) [56]	2 February – 8 May 1993	8	C	NR
Czech Republic (Olomouc and Bruntal) [56]	14 February – 2 June 1993	6	C	NR
Denmark (Randers) [57]	November 1983 – May 1984	20	C	5% (1/20)
Denmark (Hillerød municipality, Karlebo: Hørsholm and Hillerød, Frederiksborg county) [58]	January – April 1987	6	B	NR
Denmark (Hillerød municipality, Karlebo: Hørsholm and Hillerød, Frederiksborg county) [58]	August – December 1987	6	B	NR
Denmark (Hillerød municipality, Karlebo: Hørsholm and Hillerød, Frederiksborg county) [58]	1988	8	B**	NR
Denmark (Hillerød municipality, Karlebo: Hørsholm and Hillerød, Frederiksborg county) [58]	January – March 1989	6	B	NR
France (Indre-et-Loire) [59]	November 2000 – February 2002	8	B	13% (1/8)
France (Dax, Landes) [60]	December 2008 – September 2009	11	B	9% (1/11)
France (Pays de la Loire and Rhone-Alpes) [61]	12 February – 1 April 2012	16	W135	[0% (0/8)]
Germany (Rottal-Inn County) [62]	10 December 1997 – 2 March 1998	9	C	11% (1/9)
Germany (Berlin) [63]	October 2012 – May 2013	5	C	80% (4/5)
Greece (Hellenic Air Force recruit center and training base, southern Greece) [64]	19-24 January 1996	10	C	0% (0/10)
Italy (Treviso area, Veneto region, northeastern Italy) [65]	13 December 2007 – 4 January 2008	9	C	[43% (3/7)]
Italy (region NR) [66]	October 2012	4	C	0% (0/4)
The Netherlands (Zevenbergen, Klundert, Standdaarbuiten, Etten-Leur) [67]	26 July – 1 August 2001	7	C	NR
Norway (Tromsø, Northern-Norway) [68]	September – November 1981	3	B	67% (2/3)
Norway (northern Norway) [69]	1983	3	B	33% (1/3)
Poland (Skwierzyna, Lubuskie, a western province of Poland) [70]	22-24 March 2006	4	C	0% (0/4)
Poland (Goleniów and Załom in commune Goleniów and Łoźnica in commune Przybiernów, Goleniów County) [71]	10-30 March 2009	6	C	0% (0/6)
Spain (Logrofio, Rioja) [72]	13 November 1981 – 22 February 1982	11	C	18% (2/11)
Spain (Lloret de Mar, Catalonia, northeast Spain) [73]	29 January – 2 May 1996	5	B	40% (2/5)
Sweden/Scotland (across Sweden/north of Scotland) [74]	12-17 August 2015	13	W135	0% (0/13)
UK (Devon) [75]	October 1972 – May 1973	31	B	19% (6/31)
UK (region NR) [76]	16 October – 2 December 1996	7	C††	29% (2/7)
UK (Rotherham and North Nottinghamshire health districts) [77]	8 December 1995 – 16 January 1996	8	C	13% (1/8)
UK (University of Southampton) [78]	October 1997	6	C	50% (3/6)
UK (West Midlands, England) [79]	23 August – 23 September 2010	2	B	[0% (0/1)]
UK (Warwickshire area) [80]	February – June 2013	5	B	[0% (0/3)]
**South-East Asian region**				
India (Delhi) [81,82]	April – July 2005	444	C	14% (62/444)
India (Delhi) [83]	December 2005 – June 2006	531	A‡‡	[6% (15/257)]
India (Kashmir) [84]	1 February – 26 May 2006	17	A	12% (2/17)
**Eastern Mediterranean region**				
Israel (Israel Defense Force, not further specified) [85]	18-23 January 1992	3	C	NR
Israel (Israel Defense Force, not further specified) [85]	23-24 January 1992	2	C	NR
Israel (Israel Defense Force, not further specified) [85]	6-20 February 1993	3	C	NR
Saudi-Arabia (Makkah) [86]	19 March – 15 June 1992	182	A§§	[15% (15/102)]
**Western Pacific region**				
Australia (Doomadgee, northern Queensland) [87]	24 September 1990 – 11 April 1991	11	C	9% (1/11)
Australia (western Sydney, Penrith local government area) [88]	1 August – 10 September 1996	14	C	0% (0/14)
China (Jinan City) [89]	May 2010	3	C	NR
New-Zealand (Northland) [90]	10 July – 21 December 2011	13	C##	[33% (3/9)]
Taiwan (northern Taiwan) [91]	3-24 July 2017	3	NR	33% (1/3)

CFR – case-fatality rate, NR – not reported

*Fatal outcome not reported for all outbreak cases. CFR calculated for cases with known outcomes, indicated by square brackets.

†56/57 serogrouped isolates were serogroup C; 1/57 serogrouped isolates were serogroup B. CFR calculated for 56 cases with serogroup C.

‡Houck et al. reported the total number of deaths for three outbreaks combined: 9 cases died.

§8/11 serogrouped isolates were serogroup C; 1/11 serogrouped isolates were serogroup B.

#Mortality reported for laboratory and clinically confirmed cases, but not for suspected cases.

¶13/14 serogrouped isolates were serogroup B; 1/14 serogrouped isolates were serogroup A. CFR calculated for 14 confirmed cases (serogroup B and A).

**7/8 serogrouped isolates were serogroup B; 1/8 serogrouped isolates were serogroup C.

††4/5 serogrouped isolates were serogroup C; 1/5 serogrouped isolates was serogroup B.

‡‡42/195 serogrouped isolates were serogroup A; 65/195 serogrouped isolates had agglutination with combined ACYW135 antigen. CFR calculated for 257 confirmed cases.

§§Mortality reported for confirmed cases.

##9/13 serogrouped isolates were serogroup C; 4/13 serogrouped isolates were serogroup B.

### Characteristics of the patients

Age and gender were only reported for outbreaks of meningococcal disease identified in the peer-reviewed literature. There was a large variety of age ranges, and some studies only reported imprecise ranges (Table S2 in **Online Supplementary Document**). In 72% (26/36) of the outbreaks for which gender was reported, more than 50% of the cases in the outbreak were male (Table S2 in **Online Supplementary Document**).

### Characteristics of the outbreaks

**Figure 2:**
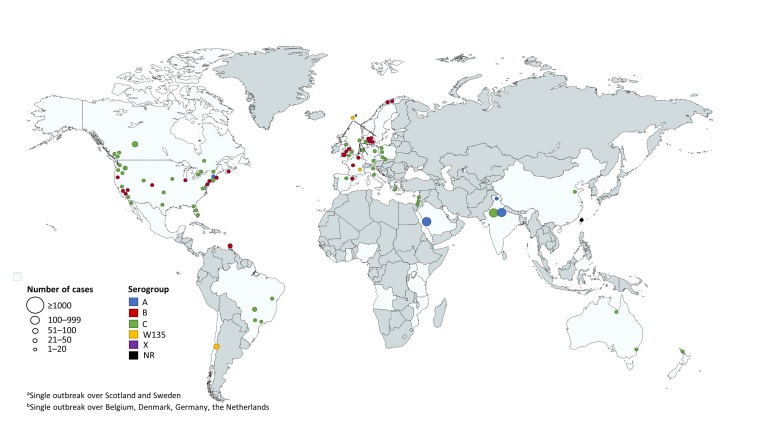
View of reported meningococcal outbreaks in non-African countries over the last 50 years.

The majority of outbreaks were identified in the regions of the Americas (41/83 outbreaks; US, 29/83; Canada, 8/83; Central and South America, 7/83), followed by the European region (30/83 outbreaks). In each of the Western Pacific, Eastern Mediterranean, and South-East Asian regions there were <10 outbreaks identified (**Figure 2** and **Table 1**). In the included outbreaks the number of cases ranged from 2 to 531, with 34% of outbreaks including >10 cases (**Table 1**); the outbreaks with the largest number of cases were seen in the South-East Asian region (**Figure 2**). Of the 54 outbreaks where CFR was calculated for all reported outbreak cases, it ranged from 0 to 80% (**Table 2**); the breakdown of CFRs by region is presented in Table S3 in **Online Supplementary Document**, however due to the small numbers of cases and the large ranges, these data should be interpreted with caution.

**Table 2:** CFR range of outbreaks of meningococcal disease in non-African countries

	CFR (%) range			
**Children**	**Adults**	**Children and adults**	**Total**
Serogroup A	NA	0.0%-11.8%	5.8%-14.7%	**0.0%-14.7%**
Serogroup B	0.0%-40.0%	0.0%-33.3%	0.0%-19.4%	**0.0%-40.0%**
Serogroup C	0.0%-50.0%	0.0%-80.0%	0.0%-66.7%	**0.0%-80.0%**
Serogroup W135	NA	NA	0.0%	**0.0%**
**All serogroups**	**0.0%-50.0%**	**0.0%-80.0%**	**0.0%-66.7%**	**0.0%-80.0%**

CFR – case-fatality rate, NA – not applicable

The most frequently reported outbreak settings were regions (such as [health] districts, areas, counties, departments, provinces; n = 20) and towns/villages/cities (n = 16); 15 outbreaks occurred in a school setting (11 of which were at universities), and other common settings included military bases (n = 9), nursery settings (n = 4), working environments (n = 3) and communities (n = 3) (Table S2 in **Online Supplementary Document**).

Serogroup C was the predominant serogroup in 61% of the outbreaks, followed by serogroup B (29%), serogroup A (5%) and serogroup W135 (4%); for one outbreak the serogroup was not reported. The timing and duration of outbreaks are visualized in Figure S1 in **Online Supplementary Document**. In the regions of the Americas between 1989 and 1993, there were multiple serogroup C outbreaks with several months duration, and a cluster of serogroup B outbreaks were reported between 2012 and 2016. In the European region, multiple serogroup B outbreaks with varying duration occurred over the last 50 years, and outbreaks of serogroup C were of short duration. Two serogroup C outbreaks in the Western Pacific region lasted for several months, while other outbreaks were of shorter duration. In the Eastern Mediterranean region all reported outbreaks, which occurred between 1992 and 1994, were of short duration and with serogroup C as the predominant serogroup. All identified outbreaks in the South-East Asian region were predominantly serogroup A and occurred after 2005.

**Figure 3:**
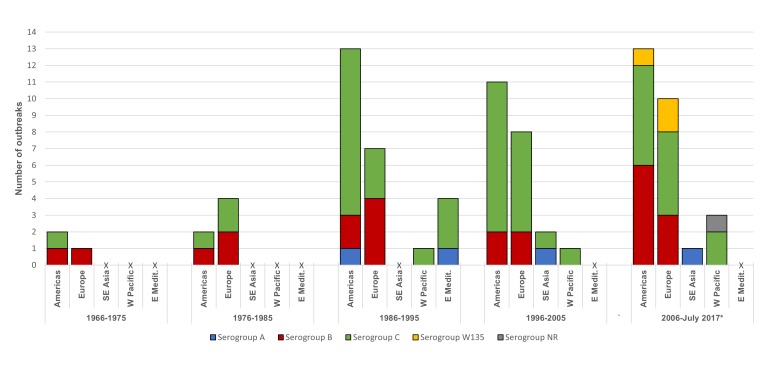
Number of outbreaks per region per 10-year period (1966-July 2017). Americas – Region of the Americas; E Medit. – Eastern Mediterranean region; Europe – European region; SE. Asia: South-East Asian region; W Pacific – Western Pacific region; X – no cases; NR – not reported. *Outbreaks in the last 1.5 years (2016-July 2017) were added to the period 2006-2015. Outbreaks which covered two 10-year periods (ie, from December 2005 to June 2006) were included in the 10-year period in which the outbreak started (n = 3).

When the occurrence of meningococcal outbreaks was examined by 10-year period, for each region it was seen that serogroup C outbreaks have been the most dominant, particularly since 1986 (**Figure 3**). Outbreaks due to serogroup B were identified in the region of the Americas and Europe over each of the 10-year periods (**Figure 3**). Low numbers of serogroup A outbreaks were reported over each period, and serogroup W135 outbreaks were only identified in the last 10 years in the region of the Americas and the European region (**Figure 3**).

**Figure 4:**
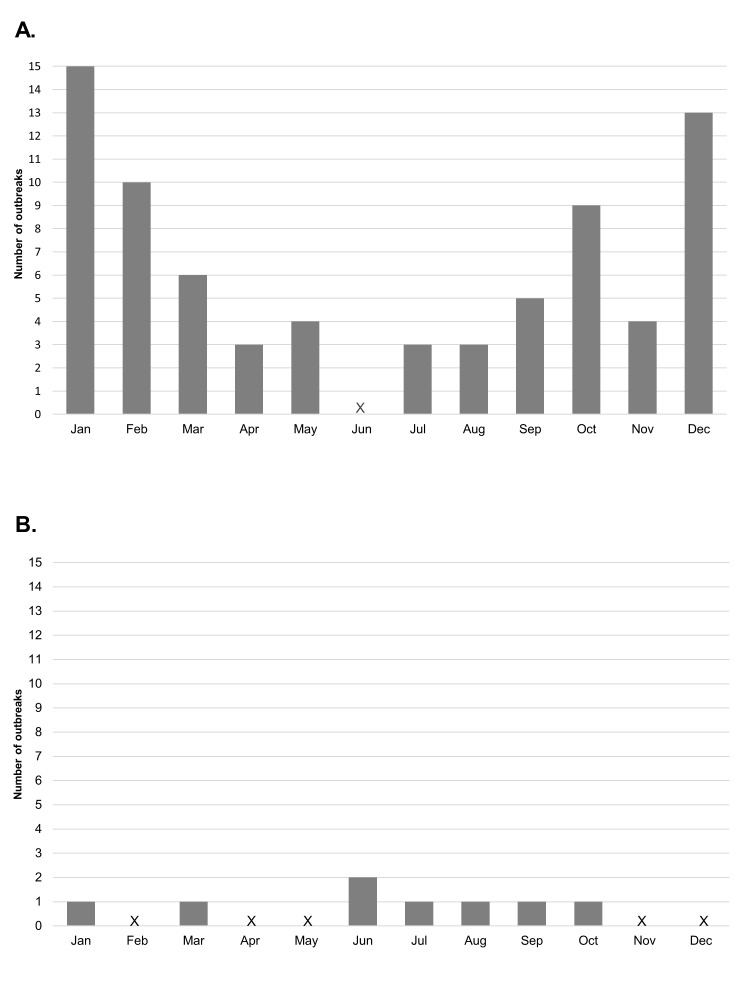
Outbreaks by month of onset in the (**A**) Northern Hemisphere and (**B**) Southern Hemisphere. X – no cases.

The seasonal pattern for the month of onset of meningococcal outbreaks showed a peak in the colder months of October, December, January and February in the Northern Hemisphere, and between June and October in the Southern Hemisphere (**Figure 4**).

## DISCUSSION

In this systematic review of meningococcal outbreaks reported over the last 50 years we show that a substantial proportion of the outbreaks identified were due to serogroup C, particularly in the regions of the Americas and Europe. Serogroup B was consistently reported in the regions of the Americas and in Europe over the last 50 years, but there appears to have been a recent increase in the number of outbreaks in the Americas region. We also identify the emergence of documented outbreaks due to serogroup W135 in recent years in South America and Europe.

Meningococcal disease patterns have been documented to vary widely over time and between geographical areas, while some of these patterns are similar to those we observed for outbreaks in this review, there are some variations. Historically, serogroup A was responsible for large epidemics across Europe, but there has been a more recent shift to the predominance of serogroup B (currently responsible for ~ 80% of cases), and for the emergence of serogroups Y and W135 [1,3,6,92]. The findings of this review differ from the pattern of epidemics, in that the predominate serogroups responsible for meningococcal disease outbreaks in Europe over the last 50 years was serogroup C, and to a lesser extent serogroup B. In the USA the incidence of meningococcal disease is low and, until recently, serogroup C was responsible for the majority of cases, with serogroup B at a consistent level (30%-40% of all disease); cases related to serogroup Y have been recently identified [1,6,7]. A similar pattern was seen in this review for outbreaks over the region of the Americas. The pattern of meningococcal disease in South America is very diverse with wide variation across countries [1,6,92]. In Asia, a shift has also been seen from a prevalence of serogroup A to serogroups B and C [1]. Many cases of meningococcal disease reported in the Eastern Mediterranean region are associated with the Hajj pilgrimage, and most recently these were due to the W135 serogroup [3]. For the South-East Asia and Eastern Mediterranean regions, the numbers of outbreaks identified in this review are too small to describe patterns of disease.

Following the introduction of vaccination, in particular with the use of conjugated vaccines in routine vaccination programs, studies of meningococcal epidemics have illustrated changes in the patterns of meningococcal disease. In Europe, serogroup B has become the dominant serogroup following widespread use of MenC vaccine from 1999 [1,6,92]. The introduction of MenB outer membrane vesicle vaccines in Cuba, Norway and New Zealand has shown to reduce the incidence of meningococcal disease caused by serogroup B [93], and the recent utilization of the new serogroup B vaccines (four-component MenB vaccine (MenB-4C) and MenB-FHbp) may act to curb outbreaks in the USA [44,94]. In Africa the introduction of the MenAfriVac MenA conjugate vaccine has radically reduced the incidence of disease caused by serogroup A, the main causative agent in the meningitis belt [95,96], This evidence demonstrates that vaccination provides a valuable resource for reducing epidemics caused by dominant vaccine serogroups and for limiting outbreaks. However, vaccination may also change the pattern of meningococcal outbreaks and epidemics, from these dominant serogroup to other serogroups, highlighting the importance of surveillance for monitoring trends in disease.

The outbreaks captured in this systematic review show a pattern of seasonality, with peaks during the winter months in both the Northern and Southern Hemispheres. This seasonality of meningococcal disease has previously been shown in the African meningitis belt, where peaks in epidemic disease occur when the humidity is at its lowest and then fall with the increase in humidity and the start of the rainy season [97]. A possible explanation is that the low humidity and dry winds damage mucosal barriers, facilitating transmission [97]. A study of the seasonal dynamics of confirmed bacterial meningitis has also shown a worldwide pattern of seasonality to infection [98].

The age groups previously identified to be at highest risk for meningococcal disease are infants, and adolescents or young adults [1,3,6]; with additional epidemiological evidence suggesting that regionally some serogroups are more common in certain age groups such as serogroup B in infants and Y in elderly populations in the USA [1,6]. Unfortunately, due to the inconsistent reporting of age in the identified publications in this review, it was not possible to draw any meaningful conclusions on the ages of those in these outbreaks. There is some conflicting evidence to suggest that male gender is a risk factor for meningococcal disease [99-101]. In this review, there was a higher proportion of males with meningococcal disease than females in the majority of included outbreaks; however, it must be taken into consideration that some settings may not have had an equal balance in gender, such as military settings, and this may have influenced these results.

One limitation to studies such as these is the limited surveillance protocols and testing facilities in some regions over time. In some countries the reporting of meningitis is not mandatory, or there is no surveillance network in place [102], consequently outbreaks of meningitis may not be documented. Some countries may also not have the facilities to accurately serogroup cases of meningococcal disease, or this may have been introduced relatively recently [102-105], consequently affecting the incidence patterns that we see here. Continuing surveillance and increasing access to testing facilities serotyping methods will further help to understand the trends in meningococcal outbreaks, as well as help identify outbreaks as they occur and take necessary action.

This systematic review identified documented meningococcal disease outbreaks reported over a 50-year period, and included a large amount of data. This allowed the examination of the temporal variation of meningococcal disease, how serogroup prevalence changed over time, and also provides a benchmark to monitor future changes in outbreaks. There are several limitations to this study, first, this review only captures outbreaks that were published in peer-reviewed publications or in the grey literature, and so the number and extent of meningococcal outbreaks will have been underestimated. There were few reports of outbreaks from the Eastern Mediterranean, Western Pacific and South-East Asia regions, which limits the generalizability of these findings. Many reports from the grey literature had no clear end date and so were excluded and a number of the articles identified could not be retrieved in full text. However, most of these articles were published in low-impact journals or concerned non-English articles. In the literature the term ‘outbreak’ was often used interchangeably with ‘epidemic’; only studies reporting on outbreaks following the definition of the CDC (same serogroup affecting a population during a shorter time period) were included. There were variations in case definitions and methods for confirmation and diagnosis for meningococcal disease, which made comparison between studies difficult. Also, the total number of deaths following the outbreak was not always reported and so CFRs were calculated based on the number of cases where the outcome was known, which may have resulted in an under- or overestimation of the true CFR. Finally, most studies included in this review concerned outbreak notifications or vaccination studies, with a limited description of the outbreak and the outbreak cases were often not studied extensively enough to draw conclusions on at-risk groups or risk factors.

This systematic review provides an overview of meningococcal disease outbreaks in several geographical areas over the last 50 years, which highlights the predominance of serogroup C and the recent emergence of W135 as the causative serogroup. The data presented here can be valuable for use in public health strategies, and monitoring future changes in outbreaks.

## Additional Material

Online Supplementary Document
