# Supplementary material for: Outbreaks of meningococcal meningitis in non-African countries over the last 50 years: a systematic review
Source: J Glob Health. 2019 Mar 26;9(1):010411. doi: 10.7189/jogh.09.010411 (PMC6441124; doi:10.7189/jogh.09.010411)
Supplement: Online Supplementary Document [file jogh-09-010411-s001.pdf]

J Glob Health 2019; 9: 010411

Online Supplementary Document

Table S1. Peer-reviewed literature and grey literature search strategy

**PubMed search terms**	#1. Meningococcal disease<br>"Meningococcal Infections"[Mesh] OR "Meningitis, Meningococcal"[Mesh] OR meningococc*[tiab]<br>AND<br>#2. Outbreaks<br>"Disease Outbreaks"[Mesh] OR outbreak*[tiab] OR epidemic*[tiab] OR pandemic*[tiab]
	Publication date January 1966 to July 2017
**EMBASE search terms**	#1. Meningococcal disease<br>'meningococcosis'/exp OR 'epidemic meningitis'/exp OR meningococc*:tiab<br>AND<br>#2. Outbreaks<br>'epidemic'/exp OR outbreak*:tiab OR epidemic*:tiab OR pandemic*:tiab
	Publication date January 1966 to July 2017
**Grey literature**	Websites were searched for information on meningococcal outbreaks using the terms 'meningococcal' and 'outbreak'<br>• ProMED mail (www.promedmail.org, search date 8 September 2017)<br>• World Health Organization (WHO) (www.who.int, search date 11 September 2017)<br>• Centers for Disease Prevention and Control (CDC) (www.cdc.gov, search date 11 September 2017)<br>• European Centre for Disease prevention and Control (ECDC) (www.ecdc.europa.eu, search date 11 September 2017)<br><br>Hits from ProMED mail were first screened based on title and abstract, followed by full-text screening. All search results on the CDC webpage, including all publications on the page of surveillance, epidemiology and outbreaks of meningococcal were screened, just as all possible relevant pages on the ECDC website that were identified using the search terms. For the WHO website, the first page of the search results and all hits on an overview page with meningococcal outbreaks worldwide.

Table S2. Characteristics of the included studies

Studies from the peer-reviewed literature

Author, year; country (region)	Study design	Study population and setting; outbreak period	Case definition	Number of cases; age; gender	*N. meningitidis* serogroup; type; subtype	Control intervention
**Region of the Americas**						
Banerji, 1995 [1]<br><br>Canada (Eastern Ontario/Western Quebec)	Outbreak investigation	MCD cases identified during outbreak in a region<br><br>December 1991–January 1992 (6 weeks)	NR	n=10<br><br>Age NR<br><br>Gender NR	Serogroup C (assumed all cases had serogroup C[1]) (type/subtype NR)	Chemoprophylaxis NR<br><br>Vaccination NR
Tyrrell, 2002 [2]<br><br>Canada (Edmonton, Alberta)	Outbreak investigation	MCD cases identified during outbreak in a region<br><br>December 1999–April 2001	Laboratory or clinically confirmed	Lab. conf.: n=57<br>Clin. conf.: n=4<br><br>Age range: 5 wks–77 yrs<br><br>Male: 47%	Serogroup C in 56/57 serogrouped isolates<br><br>Serogroup B in 1/57 serogrouped isolates (type/subtype NR)	Chemoprophylaxis (rifampin):<br>- Close contacts<br><br>Vaccination (polysaccharide quadrivalent meningococcal vaccine):<br>- Persons aged 2–19 yrs<br>- Extended in next round to all previously unimmunized 2–24 yrs
Quan, 2007 [3]<br><br>Canada (Abbotsford, British Columbia)	Follow-up study	MCD cases identified during outbreak in a village (Abbotsford) and outbreak among MSM (city NR)<br><br>Outbreak Ia: December 2000–March 2001<br><br>Outbreak Ib: October 2001–December 2001<br>Outbreak II: September–December 2004	Laboratory confirmed	Outbreak Ia: n=5<br>Outbreak Ib: n=4<br>Outbreak III: n=7<br><br>Outbreak Ia: Teenagers and young adults<br>Outbreak Ib: Older cases<br>Outbreak II: age NR<br><br>Outbreak Ia/Ib: Gender NR<br>Outbreak II: male 100%	Outbreak Ia: Serogroup C in 3/3 serogrouped isolates<br>Outbreak Ib: Serogroup C in 4/4 serogrouped isolates<br>Outbreak II: Serogroup C in 5/5 serogrouped isolates<br><br>Outbreak I, II, III (type/subtype NR)	Chemoprophylaxis NR<br><br>Vaccination NR
Tsang, 2003 [4]<br><br>Canada (Toronto, Ontario)	Outbreak investigation	MCD cases identified during outbreak among MSM in a city<br><br>Early May–mid July 2001	Laboratory confirmed	n=6<br><br>Age range: 23–39 yrs<br><br>Male: 100%	Serogroup C:NT:P1.2 in 6/6 serotyped cases	Chemoprophylaxis (type NR):<br>- Close contacts (household and potential bathhouse contacts)<br><br>Vaccination (quadrivalent polysaccharide vaccine):<br>- Homosexual or bisexual men having ≥1 risk factors
Langley, 2016 [5]<br><br>Canada (Nova Scotia)	Follow-up study	MCD cases identified during outbreak at a university	Laboratory confirmed	n=2<br><br>Age NR	Serogroup B in 2/2 serogrouped isolates (type/subtype NR)	Chemoprophylaxis NR<br><br>Vaccination (4CMenB):

Author, year; country (region)	Study design	Study population and setting; outbreak period	Case definition	Number of cases; age; gender	*N. meningitidis* serogroup; type; subtype	Control intervention
		1–11 February 2015		 Gender NR		- Students, staff, faculty
No author, 1998 [6]<br><br>USA (Florida)	Outbreak investigation	MCD cases identified during 2 outbreaks in a hotel resort and a nursing home<br><br>Outbreak I: 8 July–August 1995<br>Outbreak II: 2–10 December 1997	Outbreak I<br>Laboratory and clinically confirmed<br><br>Outbreak II<br>Laboratory confirmed	Outbreak I<br>Lab. conf.: n=4<br>Clin. conf.: n=1<br><br>Age range: up to 17 yrs<br><br>Gender NR<br><br>Outbreak II<br>n=3<br><br>Age range: 56-90 yrs<br><br>Gender NR	Outbreak I<br>Serogroup C in 4/4 serogrouped isolates (type/subtype NR)<br><br>Outbreak II<br>Serogroup C in 3/3 serogrouped isolates (type/subtype NR)	Outbreak I<br>Chemoprophylaxis (rifampin):<br>- After case 1-3, all guests and employees at both hotels<br>- After 5th case, all guests and employees at hotel A<br><br>Vaccination NR<br><br>Outbreak II<br>Chemoprophylaxis (ciprofloxacin):<br>- All persons who had visited the facility during the previous 14 days (staff members, patients, visitors)<br><br>Vaccination NR
Guttler, 1972 [7]<br><br>USA (United States Army Infantry Training Center, Fort Lewis, Washington)	Follow-up study	MCD cases identified during outbreak in military base<br><br>14 December 1970–June 1971	Laboratory confirmed	n=15<br><br>Age NR<br><br>Gender NR (assumed all cases were male[2])	Serogroup C in 15/15 serogrouped isolates (type/subtype)	Chemoprophylaxis (minocycline): - Basic combat trainees<br>- Training cadre<br>Chemoprophylaxis (minocycline): - Untreated recruits<br><br>Vaccination (group C polysaccharide vaccine):<br>- All recruits in the first 6 weeks of basic training and to all inductees entering Fort Lewis
Oill, 1978 [8]<br><br>USA (Los Angeles area, San Francisco area)	Outbreak investigation	MCD cases identified in intercity outbreak among immigrants recently arrived from Mexico<br><br>1–13 April 1974	Laboratory confirmed	n=5<br><br>Age range: 17–39 yrs<br><br>Male: 100%	Serogroup B in 5/5 serogrouped isolates (type/subtype NR)	Chemoprophylaxis (type NR):<br>- Household contacts of cases 1–3<br>Chemoprophylaxis (rifampin):<br>- Persons who lived in the residence of case 4 or who had had close contact with the patient<br><br>Vaccination NR
Brook, 1980 [9]<br><br>USA (Washington DC)	Outbreak investigation	All MCD cases identified during outbreak in a family<br><br>9 May–16 May 1979	Laboratory confirmed	n=3<br><br>Age range: 7 mo–10 yrs<br><br>Male: 33%	Serogroup B in 2/2 serogrouped isolates (type/subtype NR)	Chemoprophylaxis (rifampin for prophylaxis):<br>- All household contacts<br><br>Vaccination NR
Hudson, 1986 [10]<br><br>USA (Vermont)	Case-control study	MCD cases identified during outbreak in 2 contiguous rural counties	Laboratory confirmed	n=13<br><br>Median age: 7 yrs (range: 1–18)	Serogroup C, serogroup 4 in 1/13 serogrouped isolates<br><br>Serogroup C:2b in 12/13	Chemoprophylaxis (sulfamethoxazole):<br>- School children and their household contacts<br><br>Vaccination (meningococcal A–C vaccine):

Author, year; country (region)	Study design	Study population and setting; outbreak period	Case definition	Number of cases; age; gender	*N. meningitidis* serogroup; type; subtype	Control intervention
		15 February 1984		Male: 62%	serotyped isolates	- School students
Harrison, 1991 [11]<br><br>USA (Rockbridge County, north-western Virginia)	Case-control study	MCD cases during outbreak among school bus passenger<br><br>15–16 February 1986	Laboratory confirmed	n=5<br><br>Age range: 9–13 yrs<br><br>Gender NR	Serogroup C (number NR) (type/subtype NR)	Chemoprophylaxis (rifampin):<br>- All persons who rode the school bus<br><br>Vaccination (tetravalent A, C, W135, or Y meningococcal polysaccharide):<br>- Children of the 4 schools that had children who routinely rode the school bus
Houck, 1995 [12]<br><br>USA (Benton, Chelan, Douglas, Grant, Klickitat, and Yakima)	Outbreak investigation	MCD cases identified during outbreak in counties<br><br><u>Outbreak I</u>: 12 January–August 1989<br><u>Outbreak II</u>: September 1989–August 1990<br><u>Outbreak III</u>: September 1990–August 1991	Laboratory and clinically confirmed	<u>Outbreak I</u>: Lab. conf.: n=23. Clin. conf.: n=5<br><u>Outbreak II</u>: Lab. conf.: n=12<br><u>Outbreak III</u>: Lab. conf.: n=5<br><br>Median age: 3 yrs (range: 2 mo–77 yrs)<br><br>Gender NR	Serogroup C in 50/50 serogrouped isolates (type/subtype NR)	Chemoprophylaxis (rifampin):<br>- All case contacts<br><br>Vaccination (serogroup C meningococcal vaccination):<br>- Persons 1–24 yrs, residents of selected parts of the upper and lower valley
Morrow, 1990 [13]<br><br>USA (Santa Clara County, California)	Outbreak investigation	MCD cases identified during outbreak in a class of an intermediate school<br><br>27 January–7 February 1989	Laboratory confirmed	n=5<br><br>Age: 4/5 were students, 1/5 was a younger sibling<br><br>Gender NR	Serogroup C in 4/5 serogrouped isolates (type/subtype NR)	Chemoprophylaxis (rifampin):<br>- Entire student body and staff members of the school<br><br>Vaccination (quadrivalent (A, C, Y and W135) meningococcal vaccine):<br>- Entire student body and staff members of the school<br>- Siblings of the students
Imrey 1996 [14]<br><br>USA (region NR)	Case-control study	MCD cases identified during outbreak in a large residential university<br><br>8 February 1991–20 April 1992	Laboratory confirmed	n=9<br><br>Age range: 18–20 yrs<br><br>Male: 56%	Serogroup C in 9/9 serogrouped isolates (type/subtype NR)	Chemoprophylaxis (rifampin):<br>- Close contacts<br><br>Vaccination (quadrivalent meningococcal polysaccharide vaccine):<br>- Undergraduates and entering undergraduates
Edmond, 1995 [15]<br><br>USA (Iowa City, Iowa)	Outbreak investigation	MCD cases identified during outbreak in a university community<br><br>23 October–15 December 1992	Laboratory confirmed	n=5<br><br>Age range: 18–22 yrs<br><br>Gender NR	Serogroup C in 5/5 serogrouped isolates (type/subtype NR)	Chemoprophylaxis NR<br><br>Vaccination (tetravalent meningococcal vaccine):<br>- Student of the university
Tappero, 1996 [16]	Outbreak	MCD cases identified during	Laboratory	n=11	Serogroup C in 8/11	Chemoprophylaxis (ciprofloxacin):

Author, year; country (region)	Study design	Study population and setting; outbreak period	Case definition	Number of cases; age; gender	*N. meningitidis* serogroup; type; subtype	Control intervention
USA (Los Angeles County Men's Jail system, California)	investigation	outbreak in jail<br><br>1 January–31 March 1993	confirmed	Age NR<br><br>Male: 100%	serogrouped isolates (type/subtype NR)<br><br>Serogroup B in 1/11 serogrouped isolates (type/subtype NR)	- Inmates and workers at the jail<br><br>Vaccination NR
Wenger, 1994 [17]<br><br>USA (Grayson County, North Texas)	Outbreak investigation	MCD cases identified during outbreak in 2 neighbouring towns<br><br>24 February–21 March 1993	Laboratory confirmed	n=7<br><br>Age range: 4–32 yrs<br><br>Male: 57%	Serogroup C in 7/7 serogrouped isolates (type/subtype NR)	Chemoprophylaxis (type NR):<br>- Contacts<br><br>Vaccination (type NR):<br>- Residents aged 2–29 yrs
Jackson, 1996 [18]<br><br>USA (region NR)	Follow-up study	MCD cases identified during outbreak at a middle school<br><br>11–18 February 1995	Laboratory and clinically[3] confirmed	Lab. conf.: n=5<br>Clin. conf.: n=1<br><br>Age: students<br><br>Male: 33%	Serogroup B in 5/5 serogrouped isolates (type/subtype NR)	Chemoprophylaxis (rifampin):<br>- School students and staff<br><br>Vaccination not provided
Krause, 2002 [19]<br><br>USA (Putnam County, Florida)	Outbreak investigation	MCD cases identified during community outbreak in a county<br><br>12 December 1998–28 December 1999	Laboratory confirmed	n=12<br><br>Age range: 3 mo–25 yrs<br><br>Male: 50%	Serogroup C in 12/12 serogrouped isolates (type/subtype NR)	Chemoprophylaxis (rifampin, ciprofloxacin, or ceftriaxone):<br>- Close contacts<br><br>Mass vaccination (quadrivalent polysaccharide meningococcal vaccine):<br>- All residents aged 2–22 years of the 2 neighbouring towns<br>- Additional vaccinations to the target group but did not receive the vaccine
Schmink, 2007 [20]<br><br>USA (Chicago)	Outbreak investigation	MCD cases in MSM identified during outbreak in a city<br><br>6–15 October 2003	Laboratory confirmed	n=6<br><br>Age range: 27–42 yrs<br><br>Male: 100%	Serogroup B in 6/6 serogrouped isolates<br><br>Serogroup B:P1.5-1,10-8 in 5/5 serotyped isolates	Chemoprophylaxis (type NR):<br>- Patrons of MSM-oriented venues in the city<br><br>Vaccination (quadrivalent meningococcal polysaccharide vaccine):<br>- Patrons of MSM-oriented venues in the city
Weiss, 2009 [21]<br><br>USA (central Brooklyn, New York)	Follow-up study	MCD cases identified during outbreak among primarily illicit drug users and their contacts<br><br>1 November 2005–30 November 2006	Laboratory and clinically confirmed	Lab. conf.: n=22<br>Clin. conf.: n=1<br><br>Median age: 41 yrs (range: 4–68)<br><br>Male: 43%	Serogroup C in 22/22 serotyped isolates (type/subtype NR)	Chemoprophylaxis (type NR):<br>- Individuals who were found to be at an increased risk of infection<br><br>Vaccination (meningococcal conjugate vaccine):<br>- Adults with a history of illicit drug use or methadone use in the previous 3 months<br>- Household contacts of persons with a history of illicit drug use, living in 1 of 4 central Brooklyn zip codes

Author, year; country (region)	Study design	Study population and setting; outbreak period	Case definition	Number of cases; age; gender	*N. meningitidis* serogroup; type; subtype	Control intervention
Bradley, 2012 [22]<br><br>USA (north-eastern Oklahoma)	Outbreak investigation	All suspected MCD cases during outbreak in a school district<br><br>10 March–31 March 2010	Laboratory and clinically[5] confirmed	Lab. conf.: n=5<br>Clin. conf.: n=2<br><br>*Lab. conf. and clin. conf. without suspected cases:*<br>Age range: 5–18 yrs<br><br>Gender NR	Serogroup C in 4/4 serogrouped isolates (type/subtype NR)	Chemoprophylaxis (rifampin, intramuscular ceftriaxone injection):<br>- Students and faculty members in the lower elementary school<br>- Close contacts of the patients<br>- Extended to older students with direct contact with younger children in classrooms where cases were identified and to persons who rode on buses with the patient<br><br>Vaccination (quadrivalent meningococcal conjugate vaccine, quadrivalent meningococcal polysaccharide vaccine):<br>- All students, faculty, and staff members in the affected school district
Duffy, 2017 [23]<br><br>USA (California)	Follow-up study	MCD cases identified during outbreak at a university<br><br>March–November 2013	NR	n=5<br><br>Age: student/young adults<br><br>Gender NR	Serogroup B (numbers NR)	Chemoprophylaxis (type NR):<br>- Close contacts<br><br>Vaccination (4CMenB):<br>- Persons who were determined to be at increased risk of meningococcal serogroup B disease due to the outbreaks
McNamara, 2015 [24]<br><br>USA (New Jersey)	Follow-up study	MCD cases identified during outbreak in a university<br><br>25 March 2013–10 March 2014	Laboratory confirmed	n=9<br><br>Median age: 19 yrs (range: 17–21)<br><br>Gender: 56%	Serogroup B in 9/9 serogrouped isolates (type/subtype NR)	Chemoprophylaxis (type NR):<br>- Close contacts<br><br>Vaccination (4CMenB vaccine):<br>- Undergraduate students, graduate students living in undergraduate and graduate student dormitories, graduate students, faculty, and staff with a medical condition that increases risk of meningococcal disease, and spouses and caregivers of undergraduate and graduate students living in a dormitory with the students
Soeters, 2015 [25]<br><br>USA (Providence College, Rhode Island)	Outbreak investigation	MCD cases identified during outbreak at a college<br><br>2–5 February 2015	Laboratory confirmed	n=2<br><br>Age: both undergraduate students<br><br>Male: 100%	Serogroup B in 2/2 serogrouped isolates (type/subtype NR)	Chemoprophylaxis (ciprofloxacin):<br>- Potential contacts<br><br>Mass vaccination (MenB):<br>- Potential contacts
Biswas, 2016 [26]<br><br>USA (Santa Clara University, California)	Outbreak investigation	All MCD cases identified during outbreak at a university<br><br>31 January–2 February 2016	Laboratory and clinically[3] confirmed	Lab. conf.: n=2<br>Clin. conf.: n=1<br><br>Age: undergraduate	Serogroup B in 2/2 serogrouped isolates (type/subtype NR)	Chemoprophylaxis (ciprofloxacin):<br>- Students in social networks<br><br>Vaccination (4CMenB vaccine):

Author, year; country (region)	Study design	Study population and setting; outbreak period	Case definition	Number of cases; age; gender	*N. meningitidis* serogroup; type; subtype	Control intervention
				students<br><br>Gender NR		- All undergraduate students, graduate students and faculty and staff members at increased risk
Iser, 2012 [27]<br><br>Brazil (Rio Verde city)	Case-control study	MCD cases identified during outbreak among large food processing plant workers<br><br>June–August 2008	Laboratory or clinically[4] confirmed	Lab. conf.: n=10<br>Clin. conf.: n=12<br><br>*Lab. conf. and clin. conf. without suspected cases:*<br>Median age: 16 yrs (range: 2 mo–45 yrs)<br><br>*Lab. conf. and clin. conf. without suspected cases:*<br>Male: 63%	Serogroup C in 10/10 confirmed isolates<br><br>Serogroup C:23:P1.14–6 in 5/5 serotyped isolates	Chemoprophylaxis (type NR):<br>- Household contacts<br><br>Vaccination (polysaccharide meningococcal AC vaccine):<br>- Plant workers
Gorla, 2012 [28]<br><br>Brazil (Trancoso, Seguro, Bahia State)	Outbreak investigation	MCD cases identified during outbreak in a village<br><br>21–26 October 2009	Laboratory confirmed	n=9<br><br>Age range: 14–39 yrs<br><br>Male: 78%	Serogroup C:23:P1.5 in 5/5 serotyped isolates	Chemoprophylaxis (rifampin):<br>- Close contacts of cases<br><br>Vaccination NR
Safadi, 2014 [29]<br><br>Brazil (Cosmópolis and São José dos Campos, São Paulo State)	Cross-sectional study	MCD cases identified during 2 outbreaks in 2 oil refineries<br><br>Refinery A: 29 March–30 June 2010<br>Refinery B: 10 July–8 August 2010	Refinery A Laboratory confirmed<br><br>Refinery B NR	Refinery A: n=18<br>Refinery B: n=13<br><br>Refinery A: Age range: 8 mo – >18 yrs<br>Refinery B: <4 – >18 yrs<br><br>Gender NR	Refinery A: Serogroup C:23:P1.14-6 in 18/18 serotyped isolates<br><br>Refinery B: Serogroup C in 1/1 serogrouped isolate (assumed all cases had serogroup C[1]) (type/subtype NR)	Refinery A Chemoprophylaxis (rifampin):<br>- Close contacts<br><br>Vaccination (meningococcal A/C polysaccharide):<br>- All refinery workers<br>Mass vaccination (MCC vaccine, meningococcal A/C polysaccharide vaccine):<br>- Inhabitants of Cosmópolis, infants, toddlers, persons 2–19 yrs<br><br>Refinery B Chemoprophylaxis (type NR):<br>- Close contacts<br><br>Vaccination NR
Chacon-Cruz [30]<br><br>Mexico (Tijuana)	Outbreak investigation	MCD identified during outbreak in a city<br><br>30 January–30 March 2013	Laboratory confirmed	n=19<br><br>Median age: 16 yrs (range: 2–47)<br><br>Male: 58%	Serogroup C in 19/19 serogrouped isolates (type/subtype NR)	Chemoprophylaxis (>18 yrs: ciprofloxacin; <18 yrs: rifampin):<br>- Household contacts<br>- people who had suspected direct exposure to any confirmed case's secretion through kissing, sharing eating/drinking utensils at any time during the 7 days before onset of illness<br><br>Vaccination not provided
Chadee, 2006[31]	Outbreak	All MCD cases identified	Laboratory and	Lab. conf.: n=14	Serogroup B in 13/14	Chemoprophylaxis (rifampicin):

Author, year; country (region)	Study design	Study population and setting; outbreak period	Case definition	Number of cases; age; gender	*N. meningitidis* serogroup; type; subtype	Control intervention
Trinidad and Tobago (El Socorroi, San Juan and Claxton Bay)	investigation	during outbreak in a city<br><br>26 September–10 October 1998	clinically[3] confirmed	Clin. conf.: n=7<br><br>*Lab. conf.*:<br>Age range: <5 - >65 yrs<br><br>Gender NR	serogrouped isolates (type/subtype NR)<br><br>Serogroup A in 1/14 serogrouped isolates (type/subtype NR)	- Contacts and surveillance workers<br><br>Vaccination NR
**European region**						
Reintjes, 2002 [32]<br><br>Belgium, Denmark, Germany, The Netherlands	Outbreak investigation	MCD cases from 4 countries identified during outbreak following with a youth football tournament<br><br>9 May–24 December 1997	Laboratory confirmed	n=11<br><br>Age range: 12–39 yrs<br><br>Male: 73%	Serogroup C:2a: P1.5 in 11/11 serotyped isolates	Chemoprophylaxis (type NR):<br>- Belgium, The Netherlands, Germany: close contacts<br>- Denmark: everyone who travelled by bus<br><br>Vaccination (type NR):<br>- Belgium, Germany NR<br>- The Netherlands: town residents <20 yrs<br>- Denmark: everyone who travelled by bus
Kriz, 1995 [33]<br><br>Czech Republic (Olomouc and Bruntal)	Outbreak investigation	MCD cases identified during 2 outbreaks in 2 neighbouring districts<br><br>Olomouc: 2 February–8 May 1993<br>Bruntal: 14 February–2 June 1993	Laboratory confirmed	Olomouc: n=8<br>Bruntal: n=6<br><br>Olomouc: Age range: 2–16 yrs<br>Bruntal: Age range: 2–18 yrs<br><br>Olomouc & Bruntal: Gender NR	Olomouc & Bruntal (only reported for both outbreaks combined)<br>Serogroup C:2a:P1.2 in 7/8 serotyped isolates	Chemoprophylaxis NR<br><br>Olomouc: Mass vaccination (type NR):<br>- Students<br><br>Bruntal: Vaccination (type NR):<br>- Contacts and those requesting vaccination
Ronne, 1993 [34]<br><br>Denmark (Randers)	Follow-up study	MCD cases identified during outbreak in a city<br><br>November 1983–May 1984	Laboratory or clinically confirmed	Lab. conf.: n=19<br>Clin. conf.: n=1<br><br>Age range: 0–22 yrs<br><br>Male: 65%	Serogroup C in 19/19 serogrouped isolates<br><br>Serogroup C: 2a:P1.2 in 11/17 serotyped cases<br>Serogroup C:2a:NST in 5/17 cases<br>Serogroup C:NT:P1.2 in 1/17 case	Chemoprophylaxis (rifampicin):<br>- Classmates of second case<br><br>Vaccination after first cluster (polysaccharide vaccine A+C):<br>- All students and staff<br><br>Vaccination after 3rd cluster (type NR):<br>- All individuals attending schools or living in the area aged 10–19 yrs
Samuelsson, 1992 [35]<br><br>Denmark (Hillerød municipality, Karlebo: Hørsholm and Hillerød, Frederiksborg county)	Outbreak investigation	MCD cases identified during 4 outbreaks in various cities and communities<br><br>Outbreak I: January–April 1987<br>Outbreak II: August–	Laboratory or clinically confirmed	Outbreak I: Lab. conf.: n=5; clin. conf.: n=1<br>Outbreak II: Lab. conf.: n=5; clin. conf.: n=1<br>Outbreak III: clin. conf.: n=8<br>Outbreak IV: Lab. conf.:	Outbreak I: Serogroup B: 15: P1.16 in 5/5 serotyped isolates<br>Outbreak II: Serogroup B: 15: P1.16 in 4/5 serotyped isolates<br>Serogroup B:NT:P1.16 in 1/5	Outbreak I, II, III: Chemoprophylaxis NR<br>Outbreak IV: Chemoprophylaxis (rifampicin):<br>- All pupils and adults at schools >1 case; siblings of pupils<br><br>Outbreak I, II, III, IV: Vaccination NR

Author, year; country (region)	Study design	Study population and setting; outbreak period	Case definition	Number of cases; age; gender	*N. meningitidis* serogroup; type; subtype	Control intervention
		December 1987<br>Outbreak III: 1988<br>Outbreak IV: January–March 1989		n=5; clin. con.: n=1<br><br>Outbreak I, II, III, IV: Cases were teenagers<br><br>Outbreak I, II, III, IV: Gender NR	serotyped isolates<br>Outbreak III: Serogroup B:15:P1.16 in 7/8 serotyped isolates<br>Serogroup C:4:P1.1 in serotyped 1/8 isolates<br>Outbreak IV: Serogroup B:15:P1.16 in 5/5 serotyped isolates	
Grodet, 2004 [36]<br><br>France (Indre-et-Loire)	Outbreak investigation	MCD cases identified during outbreak in a department<br><br>November 2000–February 2002	Laboratory confirmed	n=8<br><br>Median age: 21.7 yrs (range: 14–28)<br><br>Male: 75%	Serogroup B:15:P1.12 in 8/8 serotyped isolates	Chemoprophylaxis (type NR):<br>- Family members, colleagues, friend or other people in close contact<br><br>Vaccination NR
Delisle, 2005 [37]<br><br>France (Dax, Landes)	Outbreak investigation	MCD cases identified during outbreak in a village<br><br>December 2008–September 2009	Laboratory confirmed	n=11<br><br>Median age: 18 yrs (range 7 mo–7 yrs)<br><br>Male: 55%	Serogroup B in 11/11 serogrouped isolates<br><br>Serogroup B:NT:NST in 10/10 serotyped isolates	Chemoprophylaxis (type NR):<br>- Close contacts<br>Chemoprophylaxis (rifampicin):<br>- Nightclub members<br><br>Vaccination NR
Parent du Châtelet, 2012 [38]<br><br>France (Pays de la Loire and Rhone-Alpes)	Outbreak investigation	MCD cases, recently returned from Senegal, identified during outbreak in 2 areas<br><br>12 February–1 April 2012	Laboratory confirmed	n=16<br><br>Median age: 45 yrs (range: 2 mo–89 yrs)<br><br>Male: 56%	Serogroup W135 in 16/16 serogrouped isolates<br><br>Serogroup W135:2a:P1–5,2 in 8/16 serotyped cases	Chemoprophylaxis NR<br><br>Vaccination NR
Hauri, 2000 [39]<br><br>Germany (Rottal-Inn County)	Case-control study	MCD cases identified during outbreak in a county<br><br>10 December 1997–2 March 1998	Laboratory confirmed	n=9<br><br>Age range: 2–62 yrs (1 case NR)<br><br>Gender NR	Serogroup C:2a:P1.2,5 in 9/9 serotyped isolates	Chemoprophylaxis (type NR):<br>- Close contacts<br><br>Vaccination not provided
Hellenbrand, 2016 [40]<br><br>Germany (Berlin)	Outbreak investigation	MSM cases with MCD during outbreak in a city<br><br>October 2012–May 2013	Laboratory confirmed	n=5<br><br>Age range: 20–34 yrs<br><br>Male: 100%	Serogroup C:P1.5-1,10-8:F3-6 in 5/5 serotyped isolates	Chemoprophylaxis NR<br><br>Vaccination (MenACWY):<br>- MSM
Makras, 2001 [41]<br><br>Greece (Hellenic Air Force recruit center and training base, southern	Outbreak investigation	MCD cases identified during outbreak in a military centre<br><br>19–24 January 1996	Laboratory and clinically confirmed	Lab. conf.: n=7<br>Clin. conf.: n=3<br><br>Age NR	Serogroup C in 7/7 serogrouped isolates (type/subtype NR)	Chemoprophylaxis (rifampin):<br>- Close contacts<br><br>Vaccination NR

Author, year; country (region)	Study design	Study population and setting; outbreak period	Case definition	Number of cases; age; gender	*N. meningitidis* serogroup; type; subtype	Control intervention
Greece)				Gender NR		
Ferro, 2008 [42]<br><br>Italy (Treviso area, Veneto region, north-eastern Italy)	Outbreak investigation	MCD cases identified during outbreak in an area<br><br>13 December 2007–4 January 2008	Laboratory confirmed	n=9<br><br>Mean age: 23 yrs (range: 15–33) (2 cases NR)<br><br>Gender NR	Serogroup C in 6/6 serogrouped isolates (type/subtype NR)	Chemoprophylaxis (type NR):<br>- Contacts (same places as cases)<br><br>Vaccination (type NR):<br>- 1-yr-olds and 15-yr-olds<br><br>Vaccination (meningococcal C conjugated):<br>- People aged 15–29 yrs
Van der Ende, 2003 [43]<br><br>The Netherlands (Zevenbergen, Klundert, Standdaarbuiten, Etten-Leur)	Outbreak investigation	MCD cases identified during outbreak in 4 small villages in a confined area<br><br>26 July–1 August 2001	Laboratory confirmed	n=7<br><br>Age range: 2–23 yrs<br><br>Male: 29%	Serogroup C:2a:nt in 5/7 serotyped isolates<br><br>Serogroup C:2a:P1.5 in 2/7 serotyped isolates	Chemoprophylaxis NR<br><br>Vaccination NR
Kristiansen, 1985 [44]<br><br>Norway (Tromsø, Northern-Norway)	Follow-up study	MCD cases identified during outbreak in a military camp<br><br>September–November 1981	Laboratory confirmed	n=3<br><br>Age NR<br><br>Gender NR (assumed all cases were male[2])	Serogroup B:15 in 3/3 serotyped isolates (subtype NR)	Chemoprophylaxis NR<br><br>Vaccination NR
Kristiansen, 1986 [45]<br><br>Norway (northern Norway)	Cross-sectional study	MCD cases identified during outbreak in a small community<br><br>6-week period in 1983	Laboratory confirmed	n=3<br><br>Age range: 11–12 yrs<br><br>Gender NR	Serogroup B:15 in 3/3 serotyped isolate (subtype NR)	Chemoprophylaxis NR<br><br>Vaccination NR
Grecki, 2006 [46]<br><br>Poland (Skwierzyna, Lubuskie, a western province of Poland)	Outbreak investigation	MCD cases identified during outbreak among soldiers in an army sub-unit<br><br>22–24 March 2006	Laboratory confirmed	n=4<br><br>Age NR<br><br>Gender NR (assumed all cases were male[2])	Serogroup C in 4/4 serogrouped isolates (type/subtype NR)	Chemoprophylaxis (ciprofloxacin):<br>- All soldiers of sub-unit<br>- Extended to all residents of unit, including civil personnel<br><br>Chemoprophylaxis (rifampicin):<br>- All staff member in intensive care unit<br><br>Vaccination not provided
Skoczynska, 2010 [47]<br><br>Poland (Goleniów and Załom in commune Goleniów and Łoźnica in commune Przybiernów, Goleniów County)	Outbreak investigation	MCD cases identified during outbreak in two communes<br><br>10–30 March 2009	Laboratory confirmed	n=6<br><br>Age range: 7–25 yrs<br><br>Male: 50%	Serogroup C:2a:P1.5,2 in 6/6 serotyped isolates	Chemoprophylaxis (rifampicin, cefotaxime, ciprofloxacin or azithromycin):<br>- All close contacts<br><br>Vaccination (type NR):<br>- Children aged 6–19 yrs living in affected communes, children in the same age group from other communes who attended schools in the

Author, year; country (region)	Study design	Study population and setting; outbreak period	Case definition	Number of cases; age; gender	*N. meningitidis* serogroup; type; subtype	Control intervention
						affected region, police, border guards and airport ground staff up to 24 yrs old in affected county
Saez-Nieto, 1984 [48]<br><br>Spain (Logrofio, Rioja)	Outbreak investigation	MCD cases identified during outbreak in a nursery<br><br>13 November 1981–22 February 1982	Laboratory confirmed	n=11<br><br>Age range: 2.5–12 yrs<br><br>Male: 73%	Serogroup C:2 in 3/7 serotyped isolates	Chemoprophylaxis (rifampicin):<br>- Children and all nursery staff<br>- Carriers of virulent strain after carriage study<br><br>Vaccination (polysaccharide C vaccine):<br>- Children from nursery, family contacts, adults connected to nursery
Camps, 1998 [49]<br><br>Spain (Lloret de Mar, Catalonia, northeast Spain)	Outbreak investigation	All MCD cases identified during outbreak in an indoor swimming pool<br><br>29 January–2 May 1996	Laboratory confirmed	n=5<br><br>Age range: 1–4 yrs<br><br>Male: 60%	Serogroup B in 5/5 serogrouped isolates<br><br>Serogroup B:4:P1.4 in 3/3 serotyped isolates	Chemoprophylaxis (rifampin):<br>- General chemoprophylaxis at swimming pool (not further specified)<br><br>Vaccination NR
Smith-Palmer, 2016 [50]<br><br>Scotland and Sweden	Outbreak investigation	MCD cases returning from an international event identified during outbreak<br><br>12–17 August 2015	Laboratory and clinically[5] confirmed	Lab. conf.: n=7<br>Clin. conf.: n=6<br><br>Age NR<br><br>Gender NR	Serogroup W: P1.5,2,36-2 in 6/6 serotyped isolates	Scotland<br>Chemoprophylaxis (ciprofloxacin):<br>- All scouts and leaders North of Scotland unit in addition to other close contacts of the 2 confirmed cases<br><br>Vaccination (type NR):<br>- All scouts and leaders North of Scotland unit in addition to other close contacts of the 2 confirmed cases<br><br>Sweden<br>Chemoprophylaxis (ciprofloxacin):<br>- All scouts across Sweden<br><br>Vaccination NR
Easton, 1974 [51]<br><br>UK (Devon)	Outbreak investigation	MCD cases identified during outbreak in an area<br><br>October 1972–May 1973	Laboratory or clinically confirmed	Lab. conf.: n=18<br>Clin. conf.: n=13<br><br>Age range: <1 – ≥50 yrs<br><br>Male: 55%	Serogroup B (assumed all cases had serogroup B[1])	Chemoprophylaxis (soluble sulphonamides):<br>- All contacts<br><br>Vaccination not provided
Round, 2001 [52]<br><br>UK (region NR)	Follow-up study	MCD cases identified during outbreak in university campus residents<br><br>16 October–2 December 1996	Laboratory or clinically confirmed	Lab. conf.: n=5<br>Clin. conf.: n=2<br><br>Age range: 18–20 yrs<br><br>Male: 43%	Serogroup C in 4/5 serogrouped cases<br>Serogroup B in 1/5 serogrouped cases<br><br>Serogroup C:2a: P1.5 in 2/2	Chemoprophylaxis (ciprofloxacin):<br>- All students and staff at the hall<br>- All guests who had stayed overnight in the previous 2 weeks<br><br>Vaccination (A+C meningococcal):

Author, year; country (region)	Study design	Study population and setting; outbreak period	Case definition	Number of cases; age; gender	*N. meningitidis* serogroup; type; subtype	Control intervention
					serotyped cases	- All students and staff at the hall
Irwin, 1997 [53]<br><br>UK (Rotherham and North Nottinghamshire health districts)	Follow-up study	MCD cases identified during community outbreak in 2 health districts<br><br>8 December 1995–16 January 1996	Laboratory or clinically confirmed	Lab. conf.: n=7<br>Clin. conf.: n=1<br><br>Age range: 1–17 yrs<br><br>Gender NR	Serogroup C in 7/7 serogrouped isolates<br><br>Serogroup C:2b:P1.5, P1.2 in 5/7 serotyped isolates (C:2b in 1/7 isolate)	Chemoprophylaxis (rifampicin):<br>- Primary school and younger children (<11 yrs)<br><br>Chemoprophylaxis (ciprofloxacin):<br>- Secondary school age children and young adults (aged 11–18 yrs)<br><br>Vaccination (polysaccharide):<br>- Children and young people between 2–18 years of age inclusive, living in or attending schools in the target areas
Gilmore, 1999 [54]<br><br>UK (University of Southampton)	Outbreak investigation	MCD cases identified during outbreak at a university<br><br>October, 1997	Laboratory or clinically confirmed	Lab. conf.: n=5<br>Clin. conf.: n=1<br><br>Age range: 18-19 yrs<br><br>Male: 50%	Serogroup C in 5/5 serogrouped isolates	Chemoprophylaxis (ciprofloxacin):<br>- All first-year students<br>- Extent to all first-year undergraduates and all students and staff living or working in halls of residence<br><br>Vaccination NR
Stewart, 2013 [55]<br><br>UK (West Midlands, England)	Outbreak investigation	MCD cases identified during outbreak in a nursery<br><br>23 August–23 September 2010	Laboratory confirmed	n=2<br><br>Age: 3 yrs<br><br>Gender NR	Serogroup B in 2/2 serogrouped isolates (type/subtype NR)	Chemoprophylaxis (rifampicin, ciprofloxacin):<br>- Household contacts<br>- Contacts at the nursery<br><br>Vaccination not provided
Chatt, 2014 [56]<br><br>UK (Warwickshire area)	Outbreak investigation	MCD cases identified during outbreak in a nursery<br><br>February–June 2013	Laboratory confirmed	n=5<br><br>Age range: 2 – >60 yrs<br><br>Gender NR	Serogroup B: P1.18–4,25: F1–5 in 5/5 serotyped isolates	Chemoprophylaxis (type NR):<br>- Close contacts children and staff associated with 2 of the 4 classes<br><br>Chemoprophylaxis (ciprofloxacin):<br>- All staff and children<br><br>Vaccination not provided
**South-East Asian region**						
Duggal, 2007 [57]<br><br>India (Delhi)	Outbreak investigation	MCD cases identified during outbreak in a city<br><br>December 2005–June 2006	Laboratory or clinically[3] confirmed	Lab. conf.: n=257<br>Clin. conf.: n=274<br><br>*Lab. conf.:*<br>Age range: 3 mo–65 yrs<br><br>*Lab. conf.:*<br>Male: 82%	Serogroup A in 42/195 serogrouped isolates<br><br>Agglutination with combined ACYW135 antigen in 65/195 isolates	Chemoprophylaxis NR<br><br>Vaccination NR
Kushwaha, 2010 [58]	Outbreak investigation	MCD cases identified during outbreak among soldiers	Laboratory confirmed	n=17	Serogroup A in 3/3 serogrouped isolates	Chemoprophylaxis (rifampicin):<br>- Close contacts

Author, year; country (region)	Study design	Study population and setting; outbreak period	Case definition	Number of cases; age; gender	*N. meningitidis* serogroup; type; subtype	Control intervention
India (Kashmir)		1 February–26 May 2006		Age range: 21-26 yrs (2 cases NR)<br><br>Gender NR (assumed all cases were male[2])		Vaccination not provided
Nair, 2009 [59]<br>No author, 2005 [60]<br><br>India (Delhi)	Outbreak investigation	MCD cases identified during outbreak in a city<br><br>April–July 2005	Laboratory and clinically confirmed	Lab. conf. and clin. conf: n=444 (not reported per case definition)<br><br>Age NR<br><br>Gender NR	C in 35 serogrouped isolates (total number of serogrouped isolates NR)<br>(type/subtype NR)	Chemoprophylaxis NR<br><br>Vaccination NR
**Eastern Mediterranean region, including Israel**						
Almog, 1994 [61]<br><br>Israel (Israel Defence Force, not further specified)	Outbreak investigation	MCD cases identified during 3 outbreaks among military<br><br>Outbreak I: 18–23 January 1992<br>Outbreak II: 23–24 January 1992<br>Outbreak III: 6–20 February 1993	Laboratory confirmed	Outbreak I: n=3<br>Outbreak II: n=2<br>Outbreak III: n=3<br><br>Outbreak I, II, III: Age NR<br><br>Outbreak I, II, III: Gender NR	Outbreak I: Serogroup C:NT:- in 3/3 serogrouped isolates<br><br>Outbreak II: Serogroup C:NT:- in 2/2 serogrouped isolates<br><br>Outbreak III: Serogroup C:NT:P1.2 in 3/3 serogrouped isolates	Outbreak I: Chemoprophylaxis (rifampicin):<br>- All platoon mates who shared sleeping accommodation with the case and instructors<br>- Extended to all soldiers and personnel of the company<br><br>Vaccination not provided<br><br>Outbreak II: Chemoprophylaxis (rifampicin):<br>- Cadets and teachers of 2 classes<br><br>Vaccination not provided<br><br>Outbreak III: Chemoprophylaxis (rifampicin):<br>- All recruits and personnel of the platoon and 2 medical staff personnel who treated him<br>Chemoprophylaxis (ceftriaxone)<br>- Close contacts<br><br>Vaccination not provided
Al-Gahtani, 1995 [62]<br><br>Saudi Arabia (Makkah)	Outbreak investigation	All MCD cases identified during outbreak, 3 months before the Hajj in a village<br><br>19 March–15 June 1992	Laboratory and clinically[3] confirmed	Lab. conf.: n=102<br>Clin. conf.: n=80<br><br>*Lab. conf.:*<br>Mean age: 35.3 yrs<br>*Clin. conf.:*<br>Mean age: 30.7 yrs<br><br>Lab. conf.:<br>Male: 67%	Serogroup A (assumed all lab. conf. cases had serogroup A[1])<br>(type/subtype NR)	Chemoprophylaxis NR<br><br>Mass vaccination (AC bivalent meningococcal):<br>- Residents and religious visitors who for some reason were not vaccinated in their home countries

Author, year; country (region)	Study design	Study population and setting; outbreak period	Case definition	Number of cases; age; gender	*N. meningitidis* serogroup; type; subtype	Control intervention
				Clin. conf.:<br>Male: 91%		
**Western Pacific region**						
Pearce, 1995 [63]<br><br>Australia (Doomadgee, northern Queensland)	Follow–up study	MCD cases identified during outbreak in an isolated Aboriginal community<br><br>24 September 1990–11 April 1991	Laboratory and clinically confirmed	Lab. conf. and clin. conf: n=11 (not reported per case definition)<br><br>Age range: 1–10 yrs<br><br>Male: 36%	Serogroup C in 11/11 serogrouped isolates<br><br>Serogroup C:2b:p1.2 in 5/5 serotyped isolates	Chemoprophylaxis (rifampicin):<br>- Household contacts<br><br>Mass chemoprophylaxis (rifampicin):<br>- Community<br><br>Vaccination (bivalent meningococcal polysaccharide vaccine):<br>- Children (1–15 yrs)
Jelfs, 1998 [64]<br><br>Australia (western Sydney, Penrith local government area)	Outbreak investigation	MCD cases identified during outbreak in an urban region, associated with a nightclub<br><br>1 August–10 September 1996	Laboratory and clinically[3] confirmed	Lab. conf.: n=11<br>Clin. conf.: n=3<br><br>Mean age: 23.8 yrs (range: 2–66)<br><br>Male 57%	Serogroup C:2a:P1.5 in 10/11 serotyped isolates<br><br>Serogroup C:2b:P1.5.2 in 1/11 serotyped isolate	Chemoprophylaxis (type NR):<br>- Households, close contacts, staff of nightclub and their families<br><br>Vaccination not provided
Zhang, 2013 [65]<br><br>China (Jinan City)	Follow-up study	MCD cases identified during outbreak in a jail<br><br>May 2010	Laboratory confirmed	n=3<br><br>Age range: 18–24 yrs<br><br>Male: 100%	Serogroup C in 3/3 serogrouped isolates (type/subtype NR)	Chemoprophylaxis NR<br><br>Mass vaccination (polysaccharide A+C):<br>- Close contacts
Mills, 2013 [66]<br><br>New Zealand (Northland)	Outbreak investigation	MCD cases identified during outbreak in area<br><br>10 July–21 December 2011	Laboratory confirmed	n=13<br><br><20 yrs: 89%<br><br>Gender NR	Serogroup C:2a:1.5-1,10-8 in 9/13 serotyped isolates<br><br>Serogroup B in 4/13 serogrouped isolates (type/subtype NR)	Chemoprophylaxis NR<br><br>Vaccination NR

4CMenB: multicomponent meningococcal serogroup B; Clin. conf.: clinically confirmed; Lab. conf.: laboratory confirmed; MCD: meningococcal disease; MMC: meningococcal serogroup C; mo: months; MSM: men who have sex with men; nmA: *N. meningitidis* serogroup A; NR: not reported; NST: non-sub-typeable; NT: non-typeable; UK: United Kingdom; USA: United States of America; wks: weeks; yrs: years

[1] We assumed that all cases were affected by the same serogroup (as mentioned in the article) but this was not proven by identification of the serogroup in all cases

[2] Based on the setting (e.g. military camp, MSM) it was highly likely that all cases were male

[3] All clinically confirmed cases were suspected cases as defined in the article.

[4] 6 of the 12 clinically confirmed cases were suspected cases as defined in the article

[5] All clinically confirmed cases were suspected cases as defined in the article. Although the authors decide to omit the suspected cases, this was not in line with our approach and therefore we included this cases in the table

Studies from the grey literature

Source, year; country (region)	Study population and setting; outbreak period	Number of cases; age	*N. meningitidis* serogroup[1]	Control intervention
**Region of the Americas**				
ProMED, 1998 [67]<br><br>Canada (Kitchener-Waterloo, Ontario)	MCD cases identified during outbreak in a district<br><br>3 December 1997–4 January 1998	n=7<br><br>Age range: 16–23 yrs	Serogroup C<br>(type/subtype NR)	Chemoprophylaxis NR<br><br>Vaccination (type NR):<br>- All those 2–22 yrs of age
ProMED, 1998 [68]<br><br>USA (Connecticut)	Students identified during MCD outbreak at a university<br><br>May 1993	n=3<br><br>Age: young adults	Serogroup A<br>(type/subtype NR)	Chemoprophylaxis (type NR)<br>- University of Connecticut students/staff/faculty<br><br>Vaccination NR
ProMED, 2015 [69]<br><br>USA (Eugene, Oregon)	Students identified during MCD outbreak at the university campus<br><br>13 January–19 May 2015	n=7<br><br>Age: young adults	Serogroup B<br>(type/subtype NR)	Chemoprophylaxis (type NR):<br>- 7th case's close contacts (other cases NR)<br><br>Vaccination (meningococcal serogroup B)<br>- Students
ProMED, 2012 [70]<br><br>Chile (Metropolitan Region, includes Santiago)	MCD cases identified during outbreak in a region<br><br>January–November 2013	n=46 cases (n=37 from the Santiago Metropolitan Region)<br><br>Age NR	Serogroup W135<br>(type/subtype NR)	Chemoprophylaxis NR<br><br>Vaccination (type NR)<br>- People (not further specified)
**European region**				
ProMED, 2012 [71]<br><br>Italy (region NR)	Crew members identified during MCD outbreak on a cruise ship<br><br>October 2012	n=4<br><br>Age: adults	Serogroup C<br>(type/subtype)	Chemoprophylaxis (rifampicin, ciprofloxacin):<br>- All crew members and passengers<br><br>Vaccination NR
**Western Pacific region**				
ProMED, 2017 [72]<br><br>Taiwan (northern Taiwan)	Military personnel identified during MCD outbreak at military base[2]<br><br>23–24 July 2017	n=3<br><br>Age: adults	NR	Chemoprophylaxis (type NR)<br>- All contacts<br><br>Vaccination NR

MCD: meningococcal disease; NR: not reported; USA: United States of America; yrs: years

1 We assumed that all cases were affected by the same serogroup (as mentioned in the report) but the approach of the identification of the serogroup was not clear

2 Based on the setting (e.g. military camp) it was highly likely that all cases were male

Table S3. CFR rates stratified by WHO region

Region	CFR range (%)
Region of the Americas (N=29) [1-6,9-15,17-22,24,25,27-31,67,68,73]	0.0–75.0
European region (N=23) [26,32,34,36-42,44-54,56,71]	0.0–80.0
South-East Asian region (N=3) [57-59]	5.8–14.0
Eastern Mediterranean region (N=1) [62]	14.7
Western Pacific region (N=3) [63,64,66]	0.0–33.3

CFR, case fatality rate

Figure S1. Timeline of meningococcal outbreaks in (A) the Americas region (B) the European region (C) the Western Pacific, Eastern Mediterranean, and South-East Asian region

A.

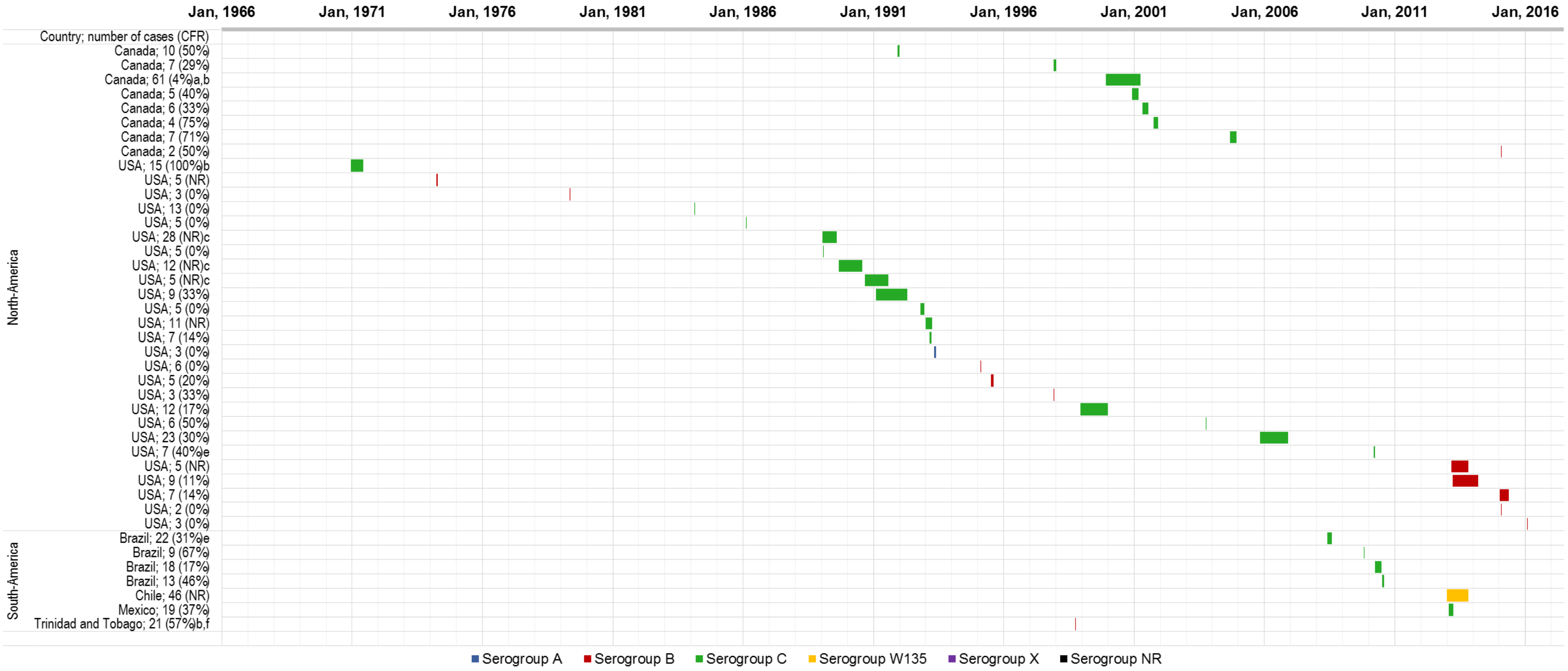

B.

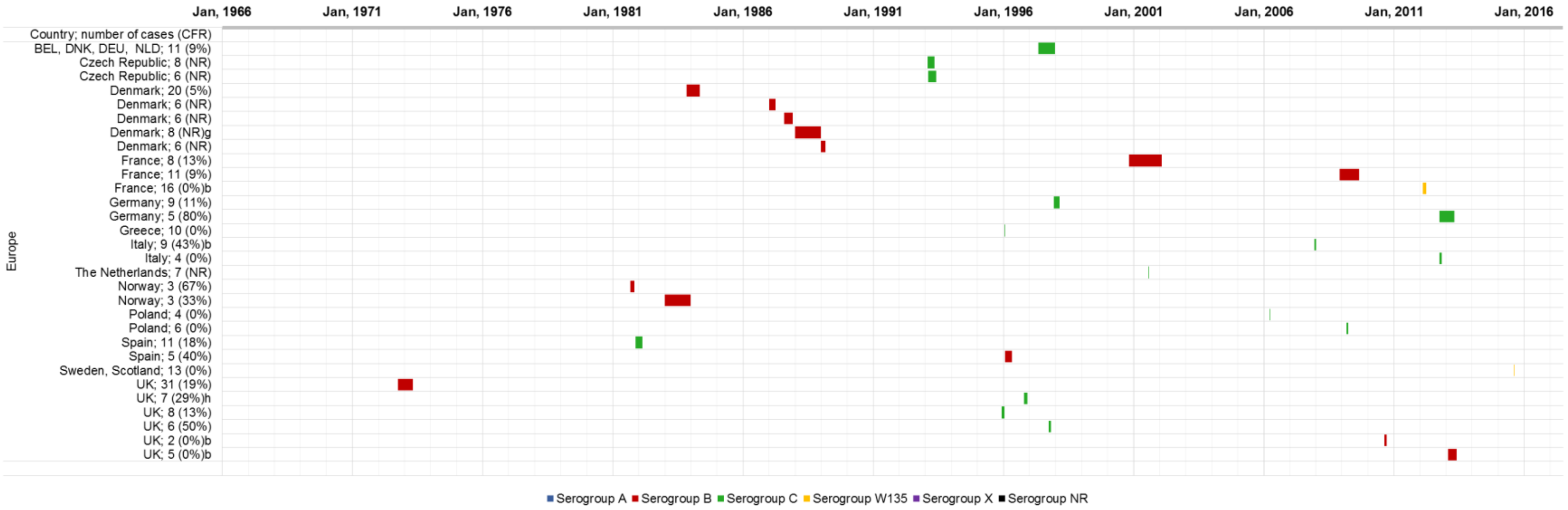
Jan, 1966
Jan, 1971
Jan, 1976
Jan, 1981
Jan, 1986
Jan, 1991
Jan, 1996
Jan, 2001
Jan, 2006
Jan, 2011
Jan, 2016
Europe
Country; number of cases (CFR)
BEL, DNK, DEU, NLD; 11 (9%)
Czech Republic; 8 (NR)
Czech Republic; 6 (NR)
Denmark; 20 (5%)
Denmark; 6 (NR)
Denmark; 6 (NR)
Denmark; 8 (NR)g
Denmark; 6 (NR)
France; 8 (13%)
France; 11 (9%)
France; 16 (0%)b
Germany; 9 (11%)
Germany; 5 (80%)
Greece; 10 (0%)
Italy; 9 (43%)b
Italy; 4 (0%)
The Netherlands; 7 (NR)
Norway; 3 (67%)
Norway; 3 (33%)
Poland; 4 (0%)
Poland; 6 (0%)
Spain; 11 (18%)
Spain; 5 (40%)
Sweden, Scotland; 13 (0%)
UK; 31 (19%)
UK; 7 (29%)h
UK; 8 (13%)
UK; 6 (50%)
UK; 2 (0%)b
UK; 5 (0%)b
Serogroup A
Serogroup B
Serogroup C
Serogroup W135
Serogroup X
Serogroup NR

C.

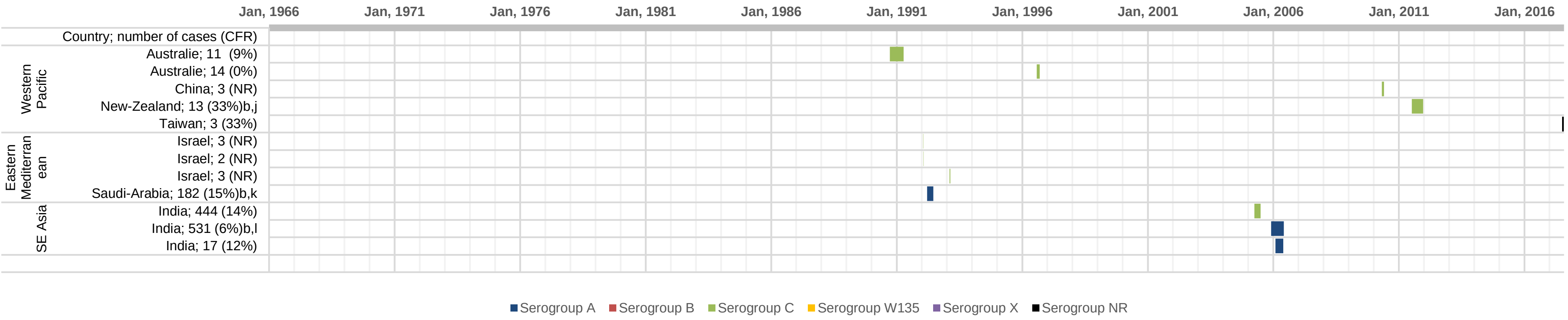

BEL: Belgium; DEU: Germany; DNK: Denmark; NLD: The Netherlands

a. Of serogrouped isolates 56/57 were serogroup C, 1/57 was serogroup B, CFR calculated over 56 cases with serogroup C; b. Fatal outcome not reported for all outbreak cases, CFR calculated over cases with known fatal outcome; c. Total number of deaths for three outbreaks combined was reported, 9 cases died; d. Of serogrouped isolates 8/11 were serogroup C, 1/11 was serogroup B; e. Mortality reported for laboratory and clinically confirmed cases; f. Of serogrouped isolates 13/14 were serogroup B, 1/14 was serogroup A, CFR calculated over 14 confirmed cases (serogroup B and A); g. Of serogrouped isolates 7/8 were serogroup B, 1/8 was serogroup C; h. Of serogrouped isolates 4/5 were serogroup C, 1/5 was serogroup B; i. Although all 6/6 serogrouped isolates were serogroup A, we want to mention the minimal number of analyses done; j. Of serogrouped isolates 9/13 were serogroup C, 4/13 were serogroup B, CFR calculated over serogroup C cases; k. Mortality reported for confirmed cases; l. Of serogrouped isolates 42/195 were serogroup A, 65/195 had agglutination with combined ACYW135 antigen, CFR calculated for confirmed cases.

References

1 Banerji A, King WJ, MacDonald N, Li M. Use of single dose ceftriaxone in the emergency department during an outbreak of serogroup C meningococcal disease. The Pediatric infectious disease journal. 1995;14:904-5.

2 Tyrrell GJ, Chui L, Johnson M, Chang N, Rennie RP, Talbot JA. Outbreak of Neisseria meningitidis, Edmonton, Alberta, Canada. Emerging infectious diseases. 2002;8:519-21.

3 Quan G, Gilbert M, David ST, Rahim T, Adie K, Shaw C, et al. Usefulness of pulsed-field gel electrophoresis in tracking two outbreaks of invasive meningococcal disease serogroup C in British Columbia. The Canadian journal of infectious diseases & medical microbiology = Journal canadien des maladies infectieuses et de la microbiologie medicale. 2007;18:363-7.

4 Tsang RS, Kiefer L, Law DK, Stoltz J, Shahin R, Brown S, et al. Outbreak of serogroup C meningococcal disease caused by a variant of Neisseria meningitidis serotype 2a ET-15 in a community of men who have sex with men. Journal of clinical microbiology. 2003;41:4411-4.

5 Langley JM, MacDougall DM, Halperin BA, Swain A, Halperin SA, Top KA, et al. Rapid surveillance for health events following a mass meningococcal B vaccine program in a university setting: A Canadian Immunization Research Network study. Vaccine. 2016;34:4046-9.

6 Outbreaks of group B meningococcal disease--Florida, 1995 and 1997. MMWR Morbidity and mortality weekly report. 1998;47:833-7.

7 Guttler RB, Beaty HN. Minocycline in the chemoprophylaxis of meningococcal disease. Antimicrobial agents and chemotherapy. 1972;1:397-402.

8 Oill PA, Chow AW, Roberto RR, Guze LB. An intercity outbreak of meningococcal meningitis in adults. The Western journal of medicine. 1978;128:189-94.

9 Brook I, Gorman R, Johnson MW. Outbreak of meningococcal disease in a family. Southern medical journal. 1980;73:902-3.

10 Hudson PJ, Vogt RL, Heun EM, Brondum J, Coffin RR, Plikaytis BD, et al. Evidence for school transmission of Neisseria meningitidis during a Vermont outbreak. Pediatric infectious disease. 1986;5:213-7.

11 Harrison LH, Armstrong CW, Jenkins SR, Harmon MW, Ajello GW, Miller GB, Jr., et al. A cluster of meningococcal disease on a school bus following epidemic influenza. Archives of internal medicine. 1991;151:1005-9.

12 Houck P, Patnode M, Atwood R, Powell K. Epidemiologic characteristics of an outbreak of serogroup C meningococcal disease and the public health response. Public health reports (Washington, DC : 1974). 1995;110:343-9.

13 Morrow HW, Slaten DD, Reingold AL, Werner SB, Fenstersheib MD. Risk factors associated with a school-related outbreak of serogroup C meningococcal disease. The Pediatric infectious disease journal. 1990;9:394-8.

14 Imrey PB, Jackson LA, Ludwinski PH, England AC, 3rd, Fella GA, Fox BC, et al. Outbreak of serogroup C meningococcal disease associated with campus bar patronage. American journal of epidemiology. 1996;143:624-30.

15 Edmond MB, Hollis RJ, Houston AK, Wenzel RP. Molecular epidemiology of an outbreak of meningococcal disease in a university community. Journal of clinical microbiology. 1995;33:2209-11.

16 Tappero JW, Reporter R, Wenger JD, Ward BA, Reeves MW, Missbach TS, et al. Meningococcal disease in Los Angeles County, California, and among men in the county jails. The New England journal of medicine. 1996;335:833-40.

17 Wenger JD, Jackson LA, Raj P, Tonelli MJ. Issues in the control of outbreaks of group C meningococcal disease in the United States. Infect Dis Clin Pract. 1994;3:136-40.

18 Jackson LA, Alexander ER, DeBolt CA, Swenson PD, Boase J, McDowell MG, et al. Evaluation of the use of mass chemoprophylaxis during a school outbreak of enzyme type 5 serogroup B meningococcal disease. The Pediatric infectious disease journal. 1996;15:992-8.

19 Krause G, Blackmore C, Wiersma S, Lesneski C, Gauch L, Hopkins RS. Mass vaccination campaign following community outbreak of meningococcal disease. Emerging infectious diseases. 2002;8:1398-403.

20 Schmink S, Watson JT, Coulson GB, Jones RC, Diaz PS, Mayer LW, et al. Molecular epidemiology of Neisseria meningitidis isolates from an outbreak of meningococcal disease among men who have sex with men, Chicago, Illinois, 2003. Journal of clinical microbiology. 2007;45:3768-70.

21 Weiss D, Stern EJ, Zimmerman C, Bregman B, Yeung A, Das D, et al. Epidemiologic investigation and targeted vaccination initiative in response to an outbreak of meningococcal disease among illicit drug users in Brooklyn, New York. Clinical infectious diseases : an official publication of the Infectious Diseases Society of America. 2009;48:894-901.

22 Bradley K, Smithee L, Clark T, Wu H, Mair R, Harcourt B, et al. Outbreak of meningococcal disease associated with an elementary school - Oklahoma, March 2010. Morbid Mortal Wkly Rep. 2012;61:217-21.

23 Duffy J, Johnsen P, Ferris M, Miller M, Leighton K, McGilvray M, et al. Safety of a meningococcal group B vaccine used in response to two university outbreaks. Journal of American college health : J of ACH. 2017:1-8.

24 McNamara LA, Shumate AM, Johnsen P, MacNeil JR, Patel M, Bhavsar T, et al. First Use of a Serogroup B Meningococcal Vaccine in the US in Response to a University Outbreak. Pediatrics. 2015;135:798-804.

25 Soeters HM, McNamara LA, Whaley M, Wang X, Alexander-Scott N, Kanadanian KV, et al. Serogroup B Meningococcal Disease Outbreak and Carriage Evaluation at a College - Rhode Island, 2015. MMWR Morbidity and mortality weekly report. 2015;64:606-7.

26 Biswas HH, Han GS, Wendorf K, Winter K, Zipprich J, Perti T, et al. Notes from the Field: Outbreak of Serogroup B Meningococcal Disease at a University - California, 2016. MMWR Morbidity and mortality weekly report. 2016;65:520-1.

27 Iser BP, Lima HC, de Moraes C, de Almeida RP, Watanabe LT, Alves SL, et al. Outbreak of Neisseria meningitidis C in workers at a large food-processing plant in Brazil: challenges of controlling disease spread to the larger community. Epidemiology and infection. 2012;140:906-15.

28 Gorla MC, de Lemos AP, Quaresma M, Vilasboas R, Marques O, de Sa MU, et al. Phenotypic and molecular characterization of serogroup C Neisseria meningitidis associated with an outbreak in Bahia, Brazil. Enfermedades infecciosas y microbiologia clinica. 2012;30:56-9.

29 Safadi MA, Carvalhanas TR, Paula de Lemos A, Gorla MC, Salgado M, Fukasawa LO, et al. Carriage rate and effects of vaccination after outbreaks of serogroup C meningococcal disease, Brazil, 2010. Emerging infectious diseases. 2014;20:806-11.

30 Chacon-Cruz E, Espinosa-De Los Monteros LE, Navarro-Alvarez S, Aranda-Lozano JL, Volker-Soberanes ML, Rivas-Landeros RM, et al. An outbreak of serogroup C (ST-11) meningococcal disease in Tijuana, Mexico. Therapeutic advances in vaccines. 2014;2:71-6.

31 Chadee DD, Lee R, Ferdinand A, Prabhakar P, Clarke D, Jacob B. Meningococcal meningitis outbreak in Trinidad, 1998. Eur J Gen Med. 2006;3:49-53.

32 Reintjes R, Kistemann T, MacLehose L, McKee M, Gill N, Weinberg J, et al. Detection and response to a meningococcal disease outbreak following a youth football tournament with teams from four European countries. International journal of hygiene and environmental health. 2002;205:291-6.

33 Kriz P, Vlckova J, Bobak M. Targeted vaccination with meningococcal polysaccharide vaccine in one district of the Czech Republic. Epidemiology and infection. 1995;115:411-8.

34 Ronne T, Berthelsen L, Buhl LH, Lind I. Comparative studies on pharyngeal carriage of Neisseria meningitidis during a localized outbreak of serogroup C meningococcal disease. Scandinavian journal of infectious diseases. 1993;25:331-9.

35 Samuelsson S, Ege P, Berthelsen L, Lind I. An outbreak of serogroup B:15:P1.16 meningococcal disease, Frederiksborg County, Denmark, 1987-9. Epidemiology and infection. 1992;108:19-30.

36 Grodet C, Dequin PF, Watt S, Lanotte P, de Gialluly C, Taha MK, et al. Outbreak in France of Neisseria meningitidis B:15:P1.12 belonging to sequence type 1403. Clinical microbiology and infection : the official publication of the European Society of Clinical Microbiology and Infectious Diseases. 2004;10:845-8.

37 Delisle E, Larrieu S, Simoes J, Laylle N, De Pommerol M, Taha MK, et al. Community outbreak of group B meningococcal disease in southwest France--December 2008 to September 2009. Euro surveillance : bulletin Europeen sur les maladies transmissibles = European communicable disease bulletin. 2010;15.

38 Parent du Chatelet I, Barboza P, Taha MK. W135 invasive meningococcal infections imported from Sub-Saharan Africa to France, January to April 2012. Euro surveillance : bulletin Europeen sur les maladies transmissibles = European communicable disease bulletin. 2012;17.

39 Hauri AM, Ehrhard I, Frank U, Ammer J, Fell G, Hamouda O, et al. Serogroup C meningococcal disease outbreak associated with discotheque attendance during carnival. Epidemiology and infection. 2000;124:69-73.

40 Hellenbrand W, Claus H, Schink S, Marcus U, Wichmann O, Vogel U. Risk of Invasive Meningococcal Disease in Men Who Have Sex with Men: Lessons Learned from an Outbreak in Germany, 2012-2013. PloS one. 2016;11:e0160126.

41 Makras P, Alexiou-Daniel S, Antoniadis A, Hatzigeorgiou D. Outbreak of meningococcal disease after an influenza B epidemic at a Hellenic Air Force recruit training center. Clinical

infectious diseases : an official publication of the Infectious Diseases Society of America. 2001;33:e48-50.

42 Ferro A, Baldo V, Cinquetti S, Corziali P, Gallo G, Lustro G, et al. Outbreak of serogroup C meningococcal disease in Veneto region, Italy. Euro surveillance : bulletin Europeen sur les maladies transmissibles = European communicable disease bulletin. 2008;13.

43 van der Ende A, Hopman CT, Keijzers WC, Spanjaard L, Lodder EB, van Keulen PH, et al. Outbreak of meningococcal disease caused by PorA-deficient meningococci. The Journal of infectious diseases. 2003;187:869-71.

44 Kristiansen BE, Sorensen B, Spanne O, Bjorvatn B. Restriction fingerprinting and serology in a small outbreak of B15 meningococcal disease among Norwegian soldiers. Scandinavian journal of infectious diseases. 1985;17:19-24.

45 Kristiansen BE, Sorensen B, Bjorvatn B, Falk ES, Fosse E, Bryn K, et al. An outbreak of group B meningococcal disease: tracing the causative strain of Neisseria meningitidis by DNA fingerprinting. Journal of clinical microbiology. 1986;23:764-7.

46 Grecki M, Bienias M. Outbreak of invasive meningococcal disease among soldiers in Skwierzyna, Poland, March 2006. Euro surveillance : bulletin Europeen sur les maladies transmissibles = European communicable disease bulletin. 2006;11:E060706.4.

47 Skoczynska A, Wasko I, Kuch A, Golebiewska A, Forys M, Hryniewicz W. Outbreak of invasive meningococcal disease in Goleniow County, northwest Poland, March 2009. Euro surveillance : bulletin Europeen sur les maladies transmissibles = European communicable disease bulletin. 2010;15.

48 Saez Nieto JA, Perucha M, Llacer A. Outbreak of meningococcal infection caused by Neisseria meningitidis Group C type 2 in a nursery. Medecine Tropicale. 1983;43:49-51.

49 Camps N, Follia N, Berrón S, De La Fuente L, Vázquez JA. An outbreak of invasive meningococcal disease probably associated with an indoor swimming pool. Clinical

microbiology and infection : the official publication of the European Society of Clinical Microbiology and Infectious Diseases. 1998;4:349-50.

50 Smith-Palmer A, Oates K, Webster D, Taylor S, Scott KJ, Smith G, et al. Outbreak of Neisseria meningitidis capsular group W among scouts returning from the World Scout Jamboree, Japan, 2015. Euro surveillance : bulletin Europeen sur les maladies transmissibles = European communicable disease bulletin. 2016;21.

51 Easton DM, Estcourt PG, Brimblecombe FS, Burgess W, Hass L, Kurtz JB. Outbreak of meningococcal disease in Devon. British medical journal. 1974;1:507-9.

52 Round A, Evans MR, Salmon RL, Hosein IK, Mukerjee AK, Smith RW, et al. Public health management of an outbreak of group C meningococcal disease in university campus residents. European journal of public health. 2001;11:431-6.

53 Irwin DJ, Miller JM, Milner PC, Patterson T, Richards RG, Williams DA, et al. Community immunization programme in response to an outbreak of invasive Neisseria meningitidis serogroup C infection in the Trent region of England 1995-1996. Journal of public health medicine. 1997;19:162-70.

54 Gilmore A, Jones G, Barker M, Soltanpoor N, Stuart JM. Meningococcal disease at the University of Southampton: outbreak investigation. Epidemiology and infection. 1999;123:185-92.

55 Stewart A, Coetzee N, Knapper E, Rajanaidu S, Iqbal Z, Duggal H. Public health action and mass chemoprophylaxis in response to a small meningococcal infection outbreak at a nursery in the West Midlands, England. Perspectives in public health. 2013;133:104-9.

56 Chatt C, Gajraj R, Hawker J, Neal K, Tahir M, Lawrence M, et al. Four-month outbreak of invasive meningococcal disease caused by a rare serogroup B strain, identified through the use of molecular PorA subtyping, England, 2013. Euro surveillance : bulletin Europeen sur les maladies transmissibles = European communicable disease bulletin. 2014;19.

57 Duggal S, Duggal N, Charoo H, Mahajan RK. Recent outbreak of meningococcal meningitis--a microbiological study with brief review of literature. The Journal of communicable diseases. 2007;39:209-16.

58 Kushwaha AS, Aggarwal SK, Arora MM. Outbreak of Meningococcal Infection amongst Soldiers Deployed in Operations. Medical journal, Armed Forces India. 2010;66:4-8.

59 Nair D, Dawar R, Deb M, Capoor MR, Singal S, Upadhayay DJ, et al. Outbreak of meningococcal disease in and around New Delhi, India, 2005-2006: a report from a tertiary care hospital. Epidemiology and infection. 2009;137:570-6.

60 Ongoing outbreak of meningococcal disease in India. Euro surveillance : bulletin Europeen sur les maladies transmissibles = European communicable disease bulletin. 2005;10:E050602.2.

61 Almog R, Block C, Gdalevich M, Lev B, Wiener M, Ashkenazi S. First recorded outbreaks of meningococcal disease in the Israel Defence Force: three clusters due to serogroup C and the emergence of resistance to rifampicin. Infection. 1994;22:69-71.

62 al-Gahtani YM, el Bushra HE, al-Qarawi SM, al-Zubaidi AA, Fontaine RE. Epidemiological investigation of an outbreak of meningococcal meningitis in Makkah (Mecca), Saudi Arabia, 1992. Epidemiology and infection. 1995;115:399-409.

63 Pearce MC, Sheridan JW, Jones DM, Lawrence GW, Murphy DM, Masutti B, et al. Control of group C meningococcal disease in Australian aboriginal children by mass rifampicin chemoprophylaxis and vaccination. Lancet (London, England). 1995;346:20-3.

64 Jelfs J, Jalaludin B, Munro R, Patel M, Kerr M, Daley D, et al. A cluster of meningococcal disease in western Sydney, Australia initially associated with a nightclub. Epidemiology and infection. 1998;120:263-70.

65 Zhang J, Zhou HJ, Xu L, Hu GC, Zhang XH, Xu SP, et al. Molecular characteristics of Neisseria meningitidis isolated during an outbreak in a jail: association with the spread and

distribution of ST-4821 complex serogroup C clone in China. Biomedical and environmental sciences : BES. 2013;26:331-7.

66 Mills C, Sexton K, Carter P. Unanswered questions, the epidemiology of a community outbreak: meningococcal C disease in Northland, New Zealand, 2011. The New Zealand medical journal. 2013;126:40-5.

67 Gold R. Meningitis, meningococcal - USA (Rhode Island) (06) ProMED mail, 1998 Contract No.: Archive Number: 19980306.0423.

68 Dardick K, Riggs, J.,. Meningitis, meningococcal - USA (Rhode Island) (02) ProMED-mail 1998 Contract No.: Archive Number: 19980302.0405.

69 Gorrow C. Meningitis, meningococcal - USA: (OR) fatal, college, sg. B, sg. B vaccine ProMED mail, 2015 Contract No.: Archive Number: 20150531.3398946.

70 Terra. Meningitis, meningococcal - Chile (04): (RM) ProMED mail, 2012 Contract No.: Archive Number: 20121118.1414639.

71 prensa-latina.cu. Meningitis, meningococcal - Italy: cruise ship, exposure ProMED mail, 2012 Contract No.: Archive Number: 20121009.1333637.

72 Taiwan Centers for Disease Control. Meningitis, meningococcal - Taiwan: fatal, military base ProMED mail, 2017 Contract No.: Archive Number: 20170801.5218924.

73 Ryan J, Douglass J. Meningitis, meningococcal - USA: (OR) college, sg B, sg B vaccine, RFI ProMED mail, 2017 Contract No.: Archive Number: 20170305.4881298.
